# M2 Macrophages Attenuate AQP2^+^ Collecting Duct Cell Apoptosis via the TRAF1–TRAF2 Complex to Suppress Randall’s Plaque Formation

**DOI:** 10.34133/research.1364

**Published:** 2026-07-16

**Authors:** Liang Tang, Zhangcheng Liao, Meng Gao, Minghui Liu, Yongchao Li, Jian Wu, Hao Yu, Cheng He, Feng Zeng, Jinbo Chen, Hequn Chen, Zewu Zhu

**Affiliations:** ^1^ Department of Urology, Xiangya Hospital, Central South University, Changsha, Hunan, China.; ^2^ National Clinical Research Center for Geriatric Disorders, Xiangya Hospital, Central South University, Changsha, Hunan, China.; ^3^ Hunan Provincial Laboratory for Diagnosis and Treatment of Genitourinary System Disease, Changsha 410008, China.; ^4^ Department of Urology, Peking Union Medical College Hospital, Chinese Academy of Medical Science and Peking Union Medical College, China.; ^5^ Institute of Physiology, University of Zurich, Zurich, Switzerland.; ^6^ Department of Internal Medicine, Section Endocrinology, Yale University School of Medicine, New Haven, CT, USA.

## Abstract

Calcium oxalate (CaOx) kidney stones are common and recur frequently, yet effective pharmacological prevention is limited. Randall’s plaques (RPs), calcium deposits on renal papillae, act as anchoring sites for CaOx crystal growth, but the cellular origin and immunoregulatory mechanism underlying early calcium deposition remain unclear. Here, we found that early calcium deposition was partially localized to the renal interstitium surrounding aquaporin 2 (AQP2)-labeled collecting duct cells with apoptotic phenotype, and inhibiting high calcium-induced apoptosis of AQP2^+^ cells markedly reduced cell layer calcium deposition. Given the regulatory role of macrophages in renal stone formation, we revealed that M2 macrophages protected AQP2^+^ cells by delivering TRAF2 via exosomes. Mechanistically, exosomal TRAF2 interacted with intracellular TRAF1 to form a stable complex, which mutually inhibited their ubiquitin-mediated degradation by excluding the shared E3 ubiquitin ligase CBLC. This complex activated downstream nuclear factor κB1 (NF-κB1) and NF-κB2 signaling. NF-κB1 enhanced TRAF1 transcription, whereas NF-κB2 increased BCL2 expression while suppressing BAX and cleaved-PARP1, thereby limiting apoptosis and subsequent calcium deposition. Based on these findings, we developed M2 exosome-loaded, AQP2^+^ cell membrane-coated poly (lactic-co-glycolic acid) nanoparticles (Exo@A-P) for renal-targeted delivery. Exo@A-P treatment reduced collecting duct apoptosis and calcium deposition in hypercalciuria and renal calcified mice (*Umod^−/−^*) without detectable toxicity, potentially supporting a targeted nanotherapeutic strategy to prevent early RP formation by utilizing TRAF2-rich exosome from M2 macrophages.

## Introduction

Kidney stone disease (nephrolithiasis) is a globally prevalent urinary disorder with an increasing clinical and socioeconomic burden. Recent large-scale epidemiological analyses and global burden studies have shown that the overall prevalence in adults approaches 10% [1,2], with substantial variation according to region, sex, climate, and metabolic comorbidities such as diabetes and metabolic syndrome [3]. Calcium-based stones remain the predominant stone type, and calcium oxalate (CaOx) stones account for approximately 80% of cases. Moreover, nearly 50% of patients experience recurrence within 5 to 10 years [4,5]. Therefore, clarifying the pathogenic mechanisms underlying CaOx stone formation is of considerable clinical significance.

With the development of endoscopy, increasing evidence supports that Randall’s plaques (RPs) serve as the nidus for most CaOx stones [6–9]. Current research suggests that RPs originate from the deposition of hydroxyapatite (HAP) crystals in the renal papillary interstitium [10]. These deposits aggregate and subsequently erode through the papillary epithelium, serving as adhesion sites for CaOx crystals in urine and thereby inducing CaOx kidney stone formation [11,12]. Various factors have been proposed to play important roles in the process of RP formation, including cell injury and death [10,13], the formation of an osteoblast-like microenvironment [13,14], and immune microenvironment imbalance [10,13]. However, the molecular mechanisms underlying renal interstitial calcium deposition remain unclear.

Clues to the origin of RP largely lie in the location of early calcium deposition, and the ascending thin loop of Henle has been traditionally considered the site of RP origin [15,16]. In contrast, we observed early calcium deposition around collecting ducts in addition to the loop of Henle within RP tissues, an observation consistent with previous studies [17,18]. These findings suggest that the role of collecting duct cells in RP origination should not be overlooked. Previous in vitro studies on CaOx stones have largely focused on proximal tubular epithelial cells [19]. Of note, HK-2 cells primarily recapitulate proximal tubular phenotypes and do not faithfully represent the biological properties of collecting duct cells. Consequently, conclusions drawn from HK-2-based models may not fully reflect the pathological processes occurring in the collecting duct microenvironment. Therefore, it is helpful to advance our understanding of RP origin to investigate collecting duct cell-associated calcium deposition. Here, we performed single-cell RNA sequencing (scRNA-seq) of RP tissues and used aquaporin 2 (AQP2), a vasopressin-regulated water channel [20], to identify collecting duct principal cells, as principal cells account for the majority of collecting duct cells [21]. Our results suggested that apoptosis of AQP2^+^ cells was involved in RP formation. Given that the renal papilla is a hypertonic environment and that hypercalciuria is a well-established risk factor for RP formation [22], we further revealed that apoptosis of AQP2^+^ cells treated by high calcium induced calcium deposition.

RP formation is driven by complex interactions among epithelial cells, immune cells, and the local interstitial microenvironment [13]. Recent studies have emphasized the critical role of the immune microenvironment in RP formation, with macrophages being a major focus of investigation [13,23]. Macrophages may exert dual effects during stone formation. M1 macrophages, activated by calcium or oxalate crystals, tend to aggravate epithelial injury and promote crystal deposition, whereas M2 macrophages are more likely to facilitate crystal clearance and tissue repair through anti-inflammatory responses [23–25]. However, the mechanisms by which macrophages influence calcium deposition remain poorly defined. Specifically, whether M2 macrophages exert additional regulatory effects on calcium deposition through alternative pathways remains largely unexplored, such as modulation of tubular epithelial cell apoptosis via exosome-mediated signaling. This represents a substantial gap in our understanding of the M2–epithelial crosstalk underlying RP formation.

Therefore, the present study aimed to investigate the crosstalk between M2 macrophages and AQP2^+^ collecting duct cells under high-calcium stress during RP formation. By clarifying this interaction, our study provides new insights into the immunopathogenesis of CaOx kidney stones and may help identify potential therapeutic targets for preventing RP-associated CaOx stone formation.

## Results

### Apoptosis of AQP2^+^ collecting duct principal cells promotes calcium deposition

**Fig. 1.**
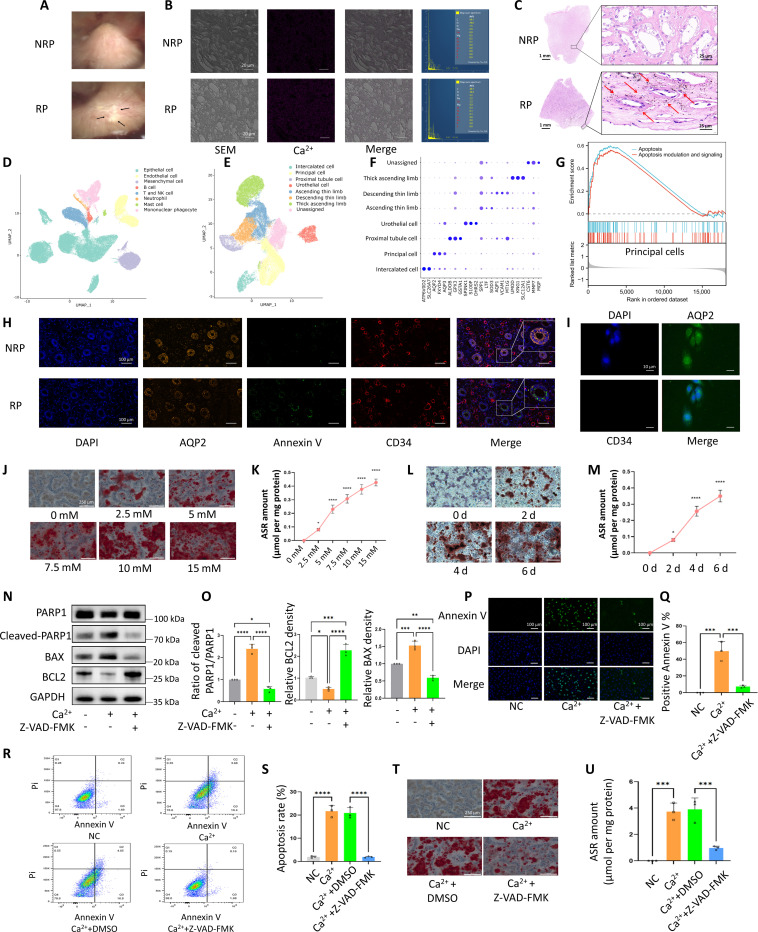
Apoptosis of AQP2^+^ collecting duct principal cells promotes calcium deposition. (A) Endoscopic appearance of normal renal papillae (NRP) and Randall’s plaque (RP) renal papillae. (B) Scanning electron microscopy (SEM) and energy-dispersive x-ray spectroscopy (EDS) elemental analysis of NRP (*n* = 3) and RP (*n* = 3) tissues. (C) Representative Von Kossa staining of RP (*n* = 6) and NRP (*n* = 6) tissues. Black arrows indicating calcium deposits. (D and E) UMAP plot of all cell clusters (D) and epithelial cell clusters (E) of single-cell transcriptomes from NRP (*n* = 5) and RP (*n* = 4) tissues. (F) Dot plot of highly variable genes across epithelial cell subpopulations. (G) GSEA enrichment analysis in apoptosis pathway of DEGs in AQP2^+^ cells from NRP and RP. (H) Representative immunofluorescent images of NRP (*n* = 6) and RP (*n* = 6) tissues showing colocalization of apoptotic regions with AQP2^+^ collecting duct principal cells. (I) Representative immunofluorescent images of primary AQP2^+^ cells confirming AQP2 expression (*n* = 3). (J and K) Alizarin Red staining of AQP2^+^ cells cultured for 6 d in media with varying calcium concentrations (0, 2.5, 5, 7.5, 10, and 15 mM) (*n* = 3). (L and M) Alizarin Red staining of AQP2^+^ cells cultured in 5 mM calcium for 0, 2, 4, and 6 d (*n* = 3). (N and O) Western blot analysis of PARP1, cleaved-PARP1, BCL2, and BAX in AQP2^+^ cells under high-calcium stimulation with or without Z-VAD-FMK treatment (20 μM; *n* = 3). (P and Q) TUNEL staining of AQP2^+^ cells cultured in high-calcium medium with or without Z-VAD-FMK treatment (20 μM; *n* = 3). (R and S) Flow cytometry analysis of AQP2^+^ cells cultured in high-calcium medium with or without Z-VAD-FMK treatment (20 μM; *n* = 3). (T and U) Alizarin Red staining of AQP2^+^ cells cultured in high-calcium medium with or without Z-VAD-FMK treatment (20 μM; *n* = 3).

In renal papillae containing RP (Fig. 1A), scanning electron microscopy (SEM) and energy-dispersive x-ray spectroscopy (EDS) analysis revealed a significantly increased calcium signal (Fig. 1B and Fig. S1A). Von Kossa staining showed significant calcium deposition around the collecting ducts within RP tissues (Fig. 1C). To clarify the mechanism of collecting duct-related calcium deposition, we performed scRNA-seq on renal papilla carrying RP (*n* = 4) and normal renal papilla (NRP) (*n* = 5) (Fig. 1D, Fig. S1B, and Table S1). Epithelial cells were identified (Fig. 1E and F), and collecting duct cells marked by AQP2 (AQP2^+^ cells) within this population were analyzed for differentially expressed genes (DEGs) (Fig. S1C and Table S2). Gene set enrichment analysis (GSEA) revealed significant enrichment of apoptotic pathways in AQP2^+^ cells from RP tissues (Fig. 1G). Immunofluorescence staining of RP tissues verified the markedly increased apoptosis of collecting duct principal cells, marked by AQP2 (Fig. 1H), suggesting that apoptosis of AQP2^+^ principal cells may be involved in RP formation.

We further isolated AQP2-labeled cells using immunomagnetic beads and confirmed the expression of the marker protein AQP2 by immunofluorescence staining (Fig. 1I). Given that hypercalciuria is a well-established risk factor for RP formation and CaOx stones [26], calcium ions were added to the culture medium to simulate the hypercalciuric state, as performed in our previous study [27]. Alizarin Red staining revealed a concentration-dependent increase in calcium deposition in AQP2^+^ cells following exposure to 0, 2.5, 5, 7.5, 10, or 15 mM calcium (Fig. 1J and K). Meanwhile, there was a time-dependent increase in calcium deposition in AQP2^+^ cells induced by 5 mM calcium medium (Fig. 1L and M). Based on these data, 5 mM calcium was selected for subsequent experiments to mimic the hypercalciuric microenvironment. Here, we identified calcium-induced apoptosis in AQP2^+^ cells, as indicated by cleaved poly (adenosine diphosphate-ribose) polymerase 1 (cleaved-PARP1), BCL2, and BAX expression, which could be rescued by the apoptosis inhibitor Z-VAD-FMK (Fig. 1N and O). This finding was corroborated by TUNEL (terminal deoxynucleotidyl transferase-mediated deoxyuridine triphosphate nick end labeling) staining (Fig. 1P and Q) and flow cytometry (Fig. 1R and S). Notably, calcium deposition induced by high-calcium treatment was significantly suppressed by Z-VAD-FMK (Fig. 1T and U). Considering that an osteogenic-like phenotype of renal tubular cells may contribute to renal interstitial calcification [28,29], we also measured osteogenic markers (RUNX2, BMP2, OCN, and OMD) following high-calcium treatment. Our results showed that these markers remained unaffected, and they were also not altered by Z-VAD-FMK treatment (Fig. S1D and E). These findings suggest that high calcium-induced apoptosis of AQP2^+^ cells contributes to RP formation.

### M2 macrophages attenuate apoptosis of AQP2^+^ cells and calcium deposition through TRAF1

**Fig. 2.**
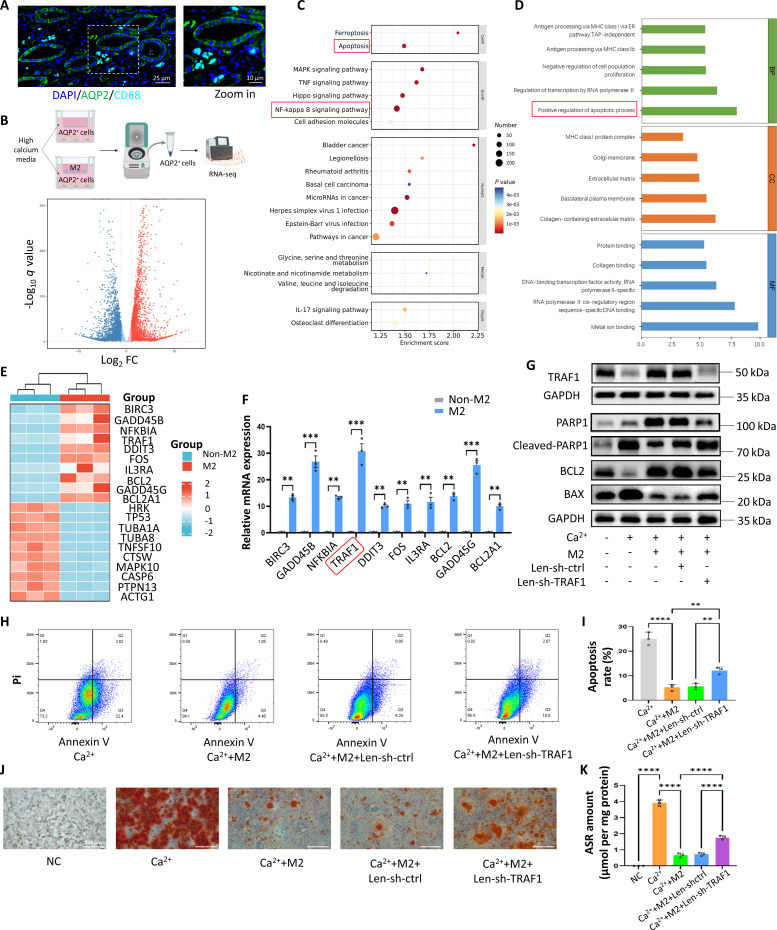
M2 macrophages attenuate apoptosis of AQP2^+^ cells and calcium deposition through TRAF1. (A) Representative images of immunofluorescence staining of AQP2 and CD68 in renal papillary tissues. (B) Transcriptomic analyses of AQP2^+^ cells cultured in high-calcium medium alone (*n* = 3) or cocultured with M2 macrophages (*n* = 3). (C and D) GO enrichment analysis (C) and KEGG pathway enrichment analysis (D) of DEGs from transcriptomic analysis. (E) Top 10 up-regulated and top 10 down-regulated genes in the “Positive regulation of apoptotic process” KEGG pathway. (F) qRT-PCR analysis of mRNA expression levels of the top 10 up-regulated genes (*n* = 3). (G to K) Western blot analysis of PARP1, cleaved-PARP1, BCL2, and BAX (G), flow cytometry analysis of apoptosis (H and I), and Alizarin Red staining of calcium deposition (J and K) in AQP2^+^ cells with *TRAF1* knockdown cocultured with M2 macrophages under high-calcium conditions. *n* = 3 for each group.

Previous studies have shown that macrophages participate in kidney stone formation and are closely associated with cellular apoptosis [30–32], and our immunofluorescence staining illustrated that macrophages were localized at the periphery of AQP2^+^ cells within the renal papillary tissues (Fig. 2A). In our study, the high calcium-induced up-regulation of cleaved-PARP1 and BCL2 expression in AQP2^+^ cells was reduced by treatment with Z-VAD-FMK or coculture with macrophages (Fig. S2A and B), indicating that macrophage coculture could attenuate calcium-induced apoptosis, as confirmed by TUNEL staining (Fig. S2C and D). To further determine whether M1 or M2 macrophages were involved, human lymphoma cells (U937) were first differentiated into adherent macrophages using phorbol myristate acetate (PMA) (100 ng/ml, 24 h). The cells were then polarized with lipopolysaccharide (LPS) (100 ng/ml) + interferon-γ (IFN-γ) (20 ng/ml) or interleukin-4 (IL-4) (20 ng/ml) for 24 h to generate M1 and M2 macrophages, respectively (Fig. S2E and F). The polarized M1 or M2 cells were subsequently cocultured with AQP2^+^ cells, which showed that calcium deposition was reduced by coculture with M2 macrophages, but not with M1 macrophages (Fig. S2G and H).

To investigate the role of M2 macrophages in AQP2^+^ cells, high-throughput transcriptomic analyses of high calcium-induced AQP2^+^ cells, with or without coculture with M2 macrophages, were conducted (Fig. 2B). DEGs from transcriptomic analysis are shown in the volcano plot (Fig. 2B and Table S3). Gene ontology (GO) (Fig. 2C) and Kyoto encyclopedia of genes and genomes (KEGG) (Fig. 2D) enrichment analyses revealed that the DEGs were markedly enriched in apoptosis-related and nuclear factor κB (NF-κB) signaling pathways. The top 10 up-regulated and down-regulated genes in the positive regulation apoptotic process (enriched KEGG pathway) were shown in Fig. 2E, among which quantitative reverse transcription polymerase chain reaction (qRT-PCR) revealed that *TRAF1* was the most strongly up-regulated expressed gene among these candidates (Fig. 2F), indicating that *TRAF1* is the potential regulator in the apoptosis of AQP2^+^ cells and calcium deposition. M2 macrophages mitigated high calcium-induced apoptosis in AQP2^+^ cells; however, this protective effect was abrogated when *TRAF1* was knocked down in AQP2^+^ cells using lentiviral transfection (Fig. 2G to I and Fig. S3A and B). Similarly, M2 macrophages suppressed high calcium-induced calcium salt deposition; however, this protective effect was abolished when *TRAF1* was knocked down in AQP2^+^ cells (Fig. 2J and K).

### M2 macrophages regulate apoptosis of AQP2^+^ cells through the TRAF1–TRAF2 interaction

**Fig. 3.**
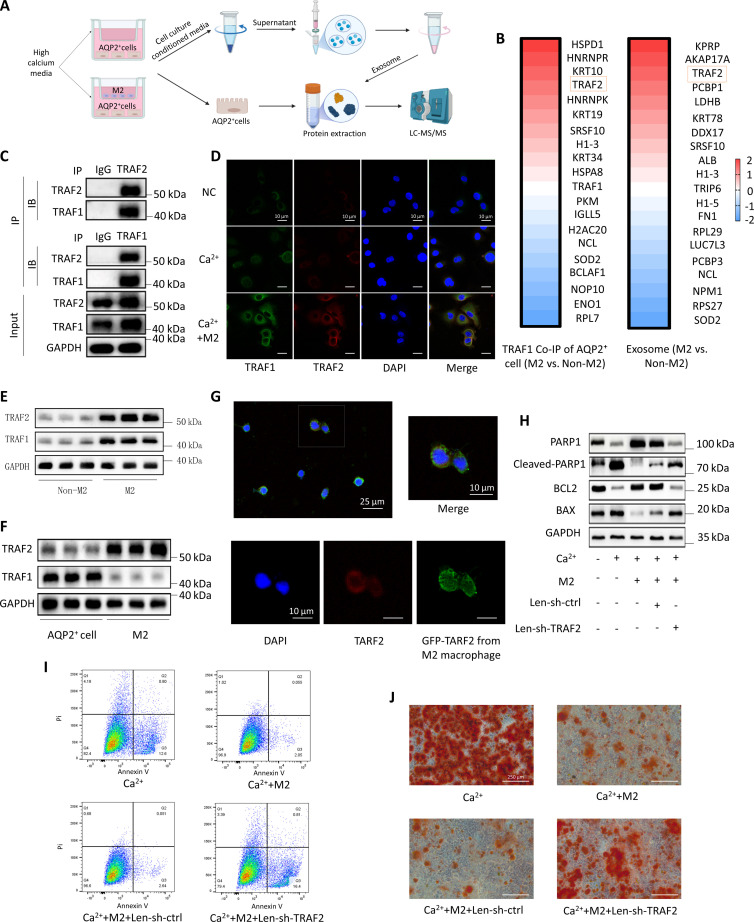
M2 macrophages regulate apoptosis of AQP2^+^ cells through the TRAF1–TRAF2 interaction. (A and B) Proteomic profiling of AQP2^+^ cells and exosomes under high-calcium conditions, comparing monoculture and M2 macrophage coculture (A) (*n* = 3). (B) Top 20 proteins by fold change (up- to down-regulated) from TRAF1 Co-IP and exosomal mass spectrometry. (C) Co-immunoprecipitation analysis of TRAF1 and TRAF2 interaction (*n* = 3). (D) Representative immunofluorescence images of TRAF1 and TRAF2 in high calcium-induced AQP2^+^ cells cocultured with M2 macrophages (*n* = 3). (E) Western blot analysis of TRAF1 and TRAF2 expression in AQP2^+^ cells under high-calcium conditions, comparing monoculture (*n* = 3) and M2 macrophage coculture (*n* = 3). (F) Western blot analysis of TRAF1 and TRAF2 expression in AQP2^+^ cells (*n* = 3) and M2 macrophages (*n* = 3), respectively. (G) M2 macrophages were transfected with the TRAF2–GFP fusion plasmid. After 48 h of coculture of AQP2^+^ cells and M2 macrophages, the fluorescence intensities of TRAF2 and GFP in AQP2^+^ cells were analyzed (*n* = 3). (H to J) Under high-calcium conditions, AQP2^+^ cells were cocultured with *TRAF2*-knockdown M2 macrophages. PARP1, cleaved-PARP1, BCL2, and BAX in AQP2^+^ cells were analyzed by Western blot (H), apoptosis by flow cytometry (I), and calcium deposition by Alizarin Red staining (J). *n* = 3 for each group.

Accumulating evidence highlights exosomes as a critical mediator of macrophage communication with neighboring cells [33], and exosome-carried proteins play an important role in intercellular communication [34]. Initially, exosomes were isolated from the supernatant of M2 macrophages, verified by exosomal markers (CD63, CD81, and TSG101) and transmission electron microscopy (Fig. S4A). DiL (1,1'-dioctadecyl-3,3,3',3'-tetramethylindocarbocyanine perchlorate)-labeled exosomes were incubated with AQP2^+^ cells, and quantitative exosome uptake assays by flow cytometry revealed that approximately 80.7% of AQP2^+^ cells had markedly internalized the exosomes (Fig. S4B). Subsequently, supernatant exosomes (Fig. 3A and Fig. S4A and B) from AQP2^+^ cells cocultured with M2 macrophages versus AQP2^+^ cells alone were analyzed by mass spectrometry (Fig. S4C and D and Tables S4 and S5), revealing 110 differentially up-regulated proteins (≥8-fold). To explore the effect of exosomes on TRAF1-binding proteins, AQP2^+^ cells cocultured with M2 macrophages or cultured alone were subjected to anti-TRAF1 co-immunoprecipitation (Co-IP) (Fig. 3A), and proteomic analysis of Co-IP proteins identified 325 differentially up-regulated proteins (≥8-fold) (Fig. S4E and F and Tables S6 and S7). The intersection of significantly differentially up-regulated proteins identified in the 2 proteomic analyses yielded 25 proteins. Among them, *TRAF2* ranked within the top 5 in both analyses (Fig. 3B), which caught our attention because TRAF1–TRAF2 heterodimer was reported to function as an important regulator of cell death processes [35]. Co-IP and immunofluorescence assays further confirmed the interaction between TRAF1 and TRAF2 (Fig. 3C and D). In addition, the expression of TRAF1 and TRAF2 increased in AQP2^+^ cells when cocultured with M2 macrophage (Fig. 3E and Fig. S4G). To clarify the origin of TRAF1 and TRAF2, the expression of TRAF1 and TRAF2 was further examined in AQP2^+^ cells and M2 macrophages separately, which showed that TRAF1 was highly expressed in AQP2^+^ cells, whereas TRAF2 was predominantly expressed in M2 macrophages (Fig. 3F and Fig. S4H). Consistently, coculture of AQP2^+^ cells and M2 macrophages carrying green fluorescent protein (GFP)–TRAF2 showed that TRAF2 staining of AQP2^+^ cells was highly colocalized with GFP–TRAF2 (Fig. 3G). These results suggest that most TRAF2 originates from M2 macrophages.

To clarify whether M2 macrophages exert a protective effect on apoptosis of AQP2^+^ cells via TRAF2, AQP2^+^ cells were cocultured with *TRAF2*-silenced M2 macrophages. The results showed that silencing of *TRAF2* (Fig. S4I) attenuated the M2-induced increase of cleaved-PARP1/PARP1 and BAX, as well as the decrease of BCL2, in AQP2^+^ cells (Fig. 3H and Fig. S4J), which was consistent with the results of flow cytometry (Fig. 3I and Fig. S4K). Moreover, silencing of *TRAF2* in M2 macrophages abolished the protective effect of M2 coculture against calcium deposition in AQP2^+^ cells (Fig. 3J and Fig. S4L). These results indicated that M2 macrophage-derived TRAF2 suppressed the apoptosis of AQP2^+^ cells to inhibit calcium deposition.

### TRAF1 and TRAF2 mutually inhibited each other’s ubiquitin-mediated degradation

**Fig. 4.**
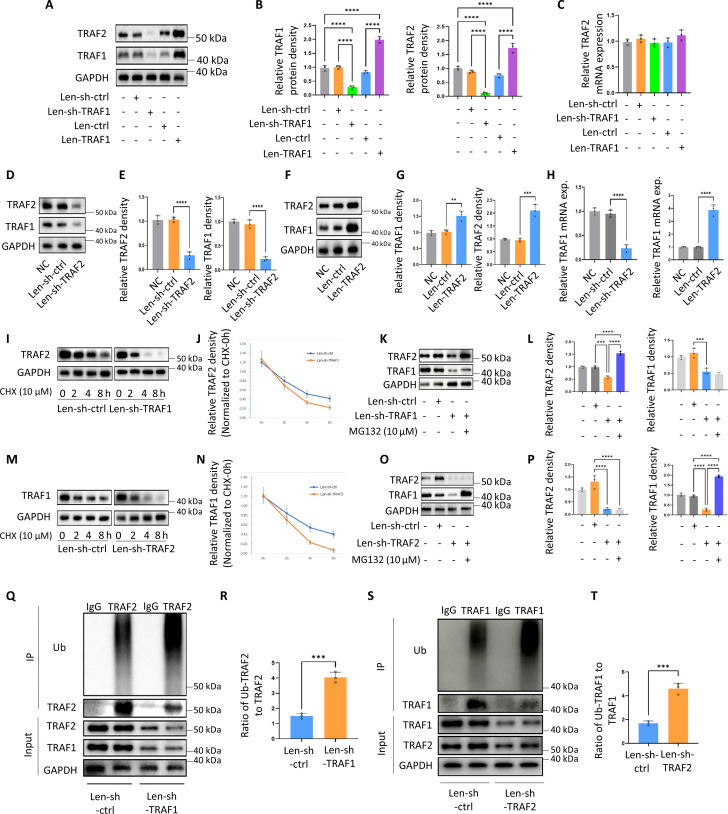
TRAF1–TRAF2 interaction forms a stable complex that promotes cooperative protein stabilization. (A to C) Western blot (A and B) and qRT-PCR (C) of TRAF2 in AQP2^+^ cells with *TRAF1* knockdown or overexpression (*n* = 3). (D to H) Western blot (D to G) and qRT-PCR (H) of *TRAF1* in AQP2^+^ cells following *TRAF2* knockdown or overexpression in M2 macrophages (*n* = 3). (I and J) Western blot of TRAF2 at 0, 2, 4, and 8 h in *TRAF1*-knockdown AQP2^+^ cells treated with 10 μM cycloheximide (CHX) (*n* = 3). (K and L) Western blot of TRAF2 in *TRAF1*-knockdown AQP2^+^ cells treated with MG132 (a proteasome inhibitor) (*n* = 3). (M and N) Western blot of TRAF1 at 0, 2, 4, and 8 h in AQP2^+^ cells treated with 10 μM CHX following *TRAF2* knockdown in M2 macrophages (*n* = 3). (O and P) Western blot of TRAF1 in AQP2^+^ cells treated with MG132 following *TRAF2* knockdown in M2 macrophages (*n* = 3). (Q and R) TRAF2 ubiquitination was assessed by Co-IP/Western blot in TRAF1-knockdown AQP2^+^ cells (*n* = 3). (S and T) TRAF1 ubiquitination was analyzed by Co-IP/Western blot in AQP2^+^ cells following *TRAF2* knockdown in M2 macrophages (*n* = 3).

Considering the interaction of TRAF1 and TRAF2 [36,37], we further explored the role of TRAF1 and TRAF2 interaction in each other’s expression. Initially, we found that *TRAF1* knockdown led to the down-regulation of TRAF2 protein, whereas *TRAF1* overexpression up-regulated the protein of TRAF2 in AQP2^+^ cells (Fig. 4A and B); however, neither manipulation affected the mRNA expression of *TRAF2* (Fig. 4C). Regarding the effect of TRAF2 on TRAF1 expression, knockdown or overexpression of *TRAF2* in M2 macrophages led to a corresponding decrease or increase of TRAF1 in AQP2^+^ cells in both protein and mRNA level (Fig. 4D to H). Given that TRAF1 and TRAF2 promote each other’s protein expression, and TRAF1 bound to TRAF2, we investigated whether TRAF1 and TRAF2 inhibit each other’s protein degradation. Utilizing cycloheximide (CHX) to inhibit protein synthesis, we revealed that knockdown of *TRAF1* significantly shortened the half-life of TRAF2 (Fig. 4I and J); similarly, knockdown of *TRAF2* significantly shortened the half-life of TRAF1 (Fig. 4M and N), suggesting reciprocal protein stabilization between TRAF1 and TRAF2. Since the ubiquitin–proteasome pathway serves as the primary route for degrading short-lived proteins [38], we hypothesized that TRAF1 and TRAF2 inhibit each other’s degradation by disrupting this system. Further experiments showed that treatment with MG-132 rescued TRAF2 degradation induced by *TRAF1* knockdown (Fig. 4K and L) and TRAF1 degradation induced by *TRAF2* knockdown (Fig. 4O and P). Subsequently, knockdown of *TRAF1* increased the ubiquitination of TRAF2 (Fig. 4Q and R), and similarly, knockdown of *TRAF2* increased the ubiquitination of TRAF1 (Fig. 4S and T). Overall, TRAF1 and TRAF2 mutually stabilize each other by protecting against ubiquitin-mediated degradation.

### TRAF1–TRAF2 interaction inhibits their ubiquitin–proteasome degradation mediated by the E3 ubiquitin ligase CBLC

**Fig. 5.**
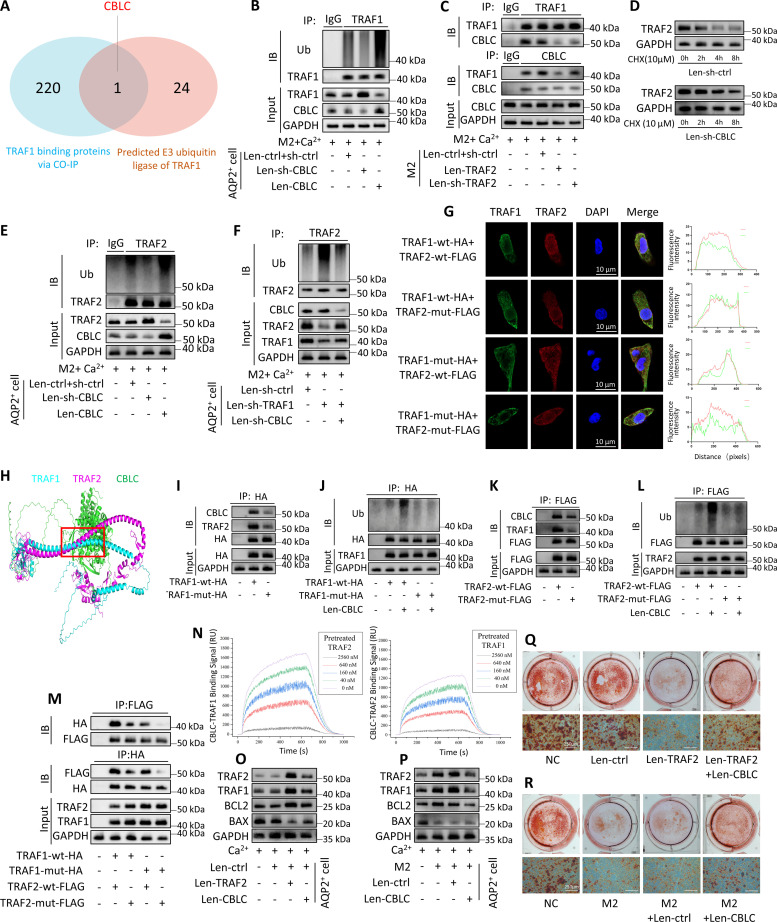
TRAF1/2 interaction inhibits CBLC-mediated proteasomal degradation. (A) Venn diagram of TRAF1-binding proteins identified by Co-IP versus predicted TRAF1 E3 ubiquitin ligases. (B) TRAF1 ubiquitination was evaluated by Co-IP/Western blot in AQP2^+^ cells of modulated CBLC cocultured with M2 macrophages (*n* = 3). (C) The interactions between CBLC and TRAF1/TRAF2 were assessed by Co-IP in AQP2^+^ cells cocultured with M2 macrophages of overexpressed or silenced *TRAF2* (*n* = 3). (D) Western blot of TRAF2 at 0, 2, 4, and 8 h in *CBLC*-knockdown AQP2^+^ cells after CHX (10 μM) treatment (*n* = 3). (E) TRAF2 ubiquitination was evaluated by Co-IP/Western blot in AQP2^+^ cells cocultured with M2 macrophages following *CBLC* overexpression or silencing (*n* = 3). (F) TRAF2 ubiquitination and degradation were evaluated by Co-IP/Western blot in AQP2^+^ cells cocultured with M2 macrophages following *TRAF1* or *CBLC* silencing (*n* = 3). (G) Immunofluorescence analysis of TRAF1/TRAF2 colocalization after individual or combined mutation of the predicted binding site of TRAF1 and TRAF2 (*n* = 3). (H) Cartoon model of the interaction interface of TRAF1, TRAF2, and CBLC predicted by AlphaFold3. The interacting amino acid residues are shown in the Supplementary Materials. (I) Interactions of TRAF1 with CBLC and TRAF2 were assessed by Co-IP/Western blot following mutation of the predicted binding site of TRAF1 (*n* = 3). (J) TRAF1 ubiquitination was evaluated by Co-IP/Western blot in AQP2^+^ cells overexpressing *CBLC* following mutation of the predicted binding sites of TRAF1 (*n* = 3). (K) Interactions of TRAF2 with CBLC and TRAF1 were assessed by Co-IP/Western blot following mutation of the predicted binding site of TRAF2 (*n* = 3). (L) TRAF2 ubiquitination was evaluated by Co-IP/Western blot in AQP2^+^ cells overexpressing *CBLC* following mutation of the predicted binding sites of TRAF2 (*n* = 3). (M) The interaction between TRAF1 and TRAF2 was evaluated by Co-IP following individual or combined mutation of the predicted binding sites of TRAF1 and TRAF2 (*n* = 3). (N) Surface plasmon resonance (SPR) assay was used to determine the interaction of CBLC with TRAF1 following treatment of the TRAF1 microarray chip with varying concentrations of TRAF2 and, similarly, the interaction of CBLC with TRAF2 following treatment of the TRAF2 microarray chip with varying concentrations of TRAF1 (*n* = 3). (O) Protein levels of BCL2 and BAX and their relative quantification were assessed in AQP2^+^ cells with TRAF2 or *CBLC* overexpression (*n* = 3). (P) BCL2 and BAX protein expression and relative quantification were assessed in AQP2^+^ cells overexpressing *CBLC* under coculture conditions with M2 macrophages (*n* = 3). (Q) Alizarin Red staining of AQP2^+^ cells with *TRAF2* or *CBLC* overexpression (*n* = 3). (R) Alizarin Red staining of AQP2^+^ cells cocultured with M2 macrophages after *CBLC* overexpression (*n* = 3).

To initially identify potential E3 ubiquitin ligases of TRAF1, we analyzed the intersection of TRAF1-binding proteins identified by TRAF1 immunoprecipitation (Table S6) and E3 ligases of TRAF1 (Table S8) predicted by UbiBrowser [39], revealing CBLC as the only overlapping candidate (Fig. 5A). Utilizing modulation of CBLC expression in AQP2^+^ cells, we verified that CBLC promoted the TRAF1 ubiquitination, and *CBLC* overexpression reversed the inhibitory effect of M2 macrophage coculture on TRAF1 ubiquitin-dependent degradation (Fig. 5B and Fig. S5A to D). To investigate whether M2-derived TRAF2 regulates TRAF1 ubiquitination by affecting the interaction between CBLC and TRAF1, Co-IP assays showed that *TRAF2* knockdown in M2 macrophages significantly increased the interaction between TRAF1 and CBLC in AQP2^+^ cells (Fig. 5C and Fig. S5E and F), whereas *TRAF2* overexpression markedly reduced this interaction. These results suggest that TRAF2 inhibits the binding of CBLC to TRAF1, thereby decreasing TRAF1 ubiquitination.

Given that our findings revealed the enhanced stability of TRAF1 and TRAF2 with each other, and CBLC is also a potential E3 ubiquitin ligase for TRAF2 (Table S9) predicted by UbiBrowser, we speculated that TRAF1 and TRAF2 might share the E3 ligase CBLC. We explored the effect of CBLC on TRAF2 stability and found that *CBLC* knockdown significantly inhibited TRAF2 degradation (Fig. 5D and Fig. S5G). Moreover, *CBLC* overexpression promoted TRAF2 ubiquitination, whereas *CBLC* silencing suppressed TRAF2 ubiquitination (Fig. 5E and Fig. S5H). Additionally, *CBLC* silencing largely abolished the TRAF2 ubiquitination promoted by *TRAF1* knockdown in AQP2^+^ cells (Fig. 5F and Fig. S5I to K).

Considering that TRAF1 and TRAF2 interaction suppresses their reciprocal ubiquitination, and that CBLC is a shared E3 ubiquitin ligase for both TRAF1 and TRAF2, we hypothesized that TRAF1–TRAF2 binding inhibits CBLC recruitment and thereby attenuates CBLC-mediated ubiquitination of TRAF1 and TRAF2. Based on AlphaFold3 structural prediction, TRAF1, TRAF2, and CBLC were found to share a common binding interface, enabling pairwise interactions (Fig. 5H and Fig. S5L to O). To test this hypothesis, a putative binding site was mutated in *TRAF1* (Table S10), which significantly reduced its interaction with both CBLC and TRAF2, and abolished *CBLC* overexpression-induced TRAF1 ubiquitination and degradation (Fig. 5G, I, and J and Fig. S5Q and R). Similarly, mutation of the corresponding site in TRAF2 (Table S10) markedly decreased its interaction with CBLC and TRAF1 and abolished CBLC-mediated TRAF2 ubiquitination and degradation (Fig. 5G, K, and L and Fig. S5S and T). Furthermore, simultaneous mutation of TRAF1 and TRAF2 further disrupted TRAF1–TRAF2 interaction (Fig. 5M and Fig. S6A and B), which was confirmed by immunofluorescence analysis (Fig. 5G and Fig. S5P). Additionally, surface plasmon resonance (SPR) assay showed that the binding affinity of CBLC for TRAF1 was inhibited by TRAF2 in a concentration-dependent manner, and similar results were observed in the affinity of CBLC for TRAF2 inhibited by TRAF1 (Fig. 5N). Collectively, these results demonstrate that CBLC, TRAF1, and TRAF2 can interact in a pairwise manner and exhibit competitive binding relationships among the 3 proteins.

As previously demonstrated, CBLC is a shared E3 ubiquitin ligase for TRAF1 and TRAF2, and the TRAF1–TRAF2 complex inhibits CBLC binding, thereby reducing ubiquitin-mediated degradation and enhancing their stability. We next investigated whether CBLC regulates the anti-apoptotic effect of M2 macrophages on AQP2^+^ cells.

Results showed that *CBLC* overexpression reversed the anti-apoptotic effect (Fig. 5O and Fig. S6C, G, and H) and reduced calcium deposition (Fig. 5Q) induced by *TRAF2* overexpression in AQP2^+^ cells. Similarly, *CBLC* overexpression also abolished the M2 macrophage-mediated protection against apoptosis (Fig. 5P and Fig. S6D to F, I, and J) and reduction in calcium deposition (Fig. 5R) in AQP2^+^ cells. Collectively, these findings indicate that CBLC, as a shared E3 ubiquitin ligase of TRAF1 and TRAF2, regulates the effects of the TRAF1–TRAF2 complex on apoptosis and calcium deposition in AQP2^+^ cells.

### M2 macrophages suppress AQP2^+^ cell apoptosis and calcium deposition via TRAF1–TRAF2-mediated activation of NF-κB1/2

**Fig. 6.**
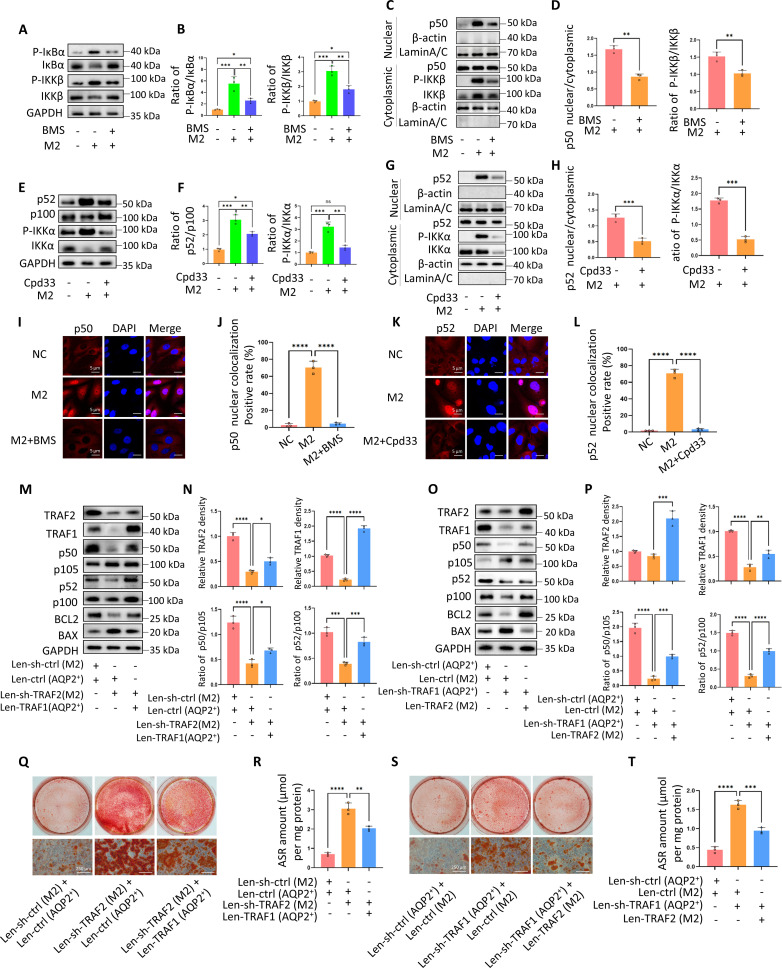
M2 macrophages suppress AQP2^+^ cell apoptosis and calcium deposition via TRAF1–TRAF2-mediated activation of NF-κB1/2. (A and B) Western blot of IκBα, IKKβ, p-IκBα, and p-IKKβ with or without BMS-345541 hydrochloride (BMS, a selective inhibitor of the catalytic subunits of IKK) treatment (*n* = 3). (C and D) Western blot analysis of p50 (cytoplasmic/nuclear) and cytoplasmic IKKβ and p-IKKβ with or without BMS treatment (*n* = 3). (E and F) Western blot of p52, p100, IKKα, and p-IKKα with or without Cpd33 treatment (*n* = 3). (G and H) Western blot analysis of p52 (cytoplasmic/nuclear) and cytoplasmic IKKα and p-IKKα with or without Cpd33 treatment (*n* = 3). (I and J) Immunofluorescence of p50 nuclear translocation in AQP2^+^ cells with or without BMS treatment (*n* = 3). (K and L) Immunofluorescence of p52 nuclear translocation in AQP2^+^ cells with or without NF-κB2 inhibitor Cpd33 (*n* = 3). (M and N) Western blot of p50, p52, BCL2, and BAX in AQP2^+^ cells following *TRAF2* overexpression in M2 macrophage, with or without *TRAF1* overexpression (*n* = 3). (O and P) Western blot of p50, p52, BCL2, and BAX in *TRAF1*-knockdown AQP2^+^ cells, with or without *TRAF2* overexpression in M2 macrophage (*n* = 3). (Q and R) Alizarin Red staining of *TRAF1*-knockdown AQP2^+^ cells following *TRAF2* overexpression in M2 macrophage, with or without *TRAF1* overexpression (*n* = 3). (S and T) Alizarin Red staining of AQP2^+^ cells in *TRAF1*-knockdown AQP2^+^ cells, with or without *TRAF2* overexpression in M2 macrophage (*n* = 3).

We further investigated the potential downstream pathways of TRAF1 and TRAF2 through which M2 macrophages protect AQP2^+^ cells from apoptosis and calcium deposition. KEGG enrichment analysis indicated that AQP2^+^ cells cocultured with M2 macrophages were markedly enriched in the NF-κB signaling pathway (Fig. 2C). The NF-κB signaling pathway consists of the canonical pathway (NF-κB1, mainly mediated by p50/RelA) and the noncanonical pathway (NF-κB2, mainly mediated by p52/RelB), both of which regulate inflammation, cell survival, and apoptosis [40,41]. Our findings showed the increased ratios of p-IKKβ/IKKβ (inhibitor of nuclear factor κB kinase subunit β) and p-IκBα/IκBα (inhibitor of nuclear factor κBα) by coculture (Fig. 6A and B), along with enhanced nuclear translocation of p50 (Fig. 6C and D), which was further confirmed by immunofluorescence staining (Fig. 6I and J), indicating activation of the NF-κB1 pathway. This activation was reversed by BMS-345541 hydrochloride (Fig. 6A to D, I, and J), a selective NF-κB1 pathway inhibitor. Similarly, coculture with M2 macrophages led to significant increases in the ratios of p52/p100 and p-IKKα/IKKα (inhibitor of nuclear factor κB kinase subunit α) (Fig. 6E and F), accompanied by enhanced nuclear translocation of p52 (Fig. 6G, H, K, and L), confirming activation of the NF-κB2 pathway, which was reversed by Cpd33, a selective NF-κB2 pathway inhibitor (Fig. 6E to H, K, and L).

TRAF1 and TRAF2 were reported to interact with each other and cooperatively regulate NF-κB signaling [42]. Knockdown of *TRAF2* in M2 macrophages reduced p50/p105 and p52/p100 levels (Fig. 6M and N), indicating inhibition of both the NF-κB1 and NF-κB2 pathways. This was accompanied by decreased BCL2 and increased BAX in AQP2^+^ cells (Fig. 6M and N), aligning to the increased calcium deposition (Fig. 6Q and R). Notably, this phenomenon was rescued by the overexpression of TRAF1 in AQP2^+^ cells (Fig. 6M, N, Q, and R). On the other hand, knockdown of TRAF1 in AQP2^+^ cells reduced p50/p105 and p52/p100 levels (Fig. 6O and P), indicating suppression of both the NF-κB1 and NF-κB2 pathways. This was accompanied by decreased BCL2 and increased BAX (Fig. 6O and P), consistent with enhanced calcium deposition (Fig. 6S and T). Importantly, overexpression of *TRAF2* in M2 macrophages rescued the suppressed NF-κB1/2 signaling and attenuated apoptosis in AQP2^+^ cells (Fig. 6O, P, S, and T). Taken together, these results suggested that M2 macrophages suppress AQP2^+^ cell apoptosis and calcium deposition via TRAF1–TRAF2-mediated activation of NF-κB1/2.

### Activation of the NF-κB1 pathway promotes the transcription of TRAF1

**Fig. 7.**
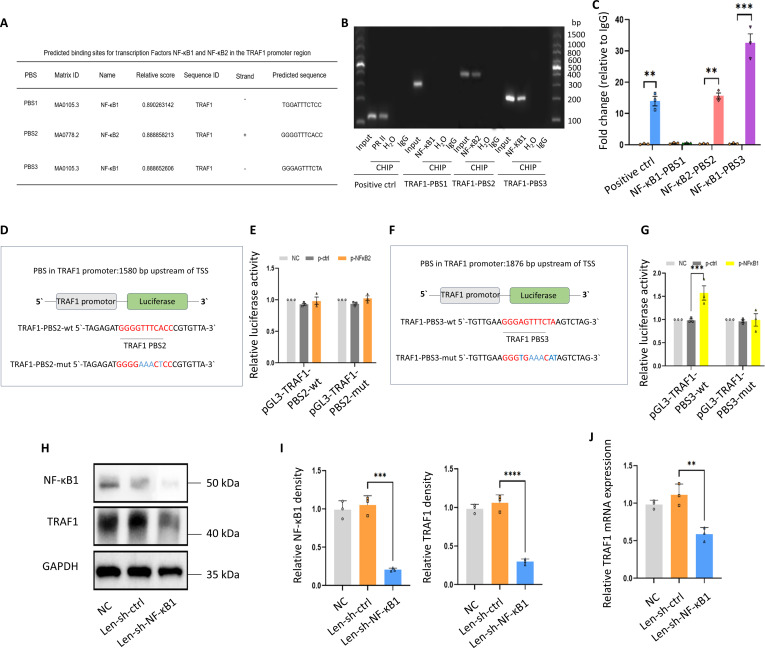
Activation of the NF-κB1 pathway promotes the transcription and translation of TRAF1. (A) Predicted binding sites for transcription factors NF-κB1 and NF-κB2 in the *TRAF1* promoter region. (B and C) Chromatin immunoprecipitation (ChIP) assay with qPCR and agarose gel electrophoresis detecting DNA fragments bound to NF-κB1 (*n* = 3) and NF-κB2 (*n* = 3). (D to G) Dual-luciferase reporter assay identifying transcription factor binding sites and functional transcription factors in the *TRAF1* promoter region (*n* = 3). (H to J) TRAF1 expression in *NF-κB1*-knockdown AQP2^+^ cells analyzed by Western blot (H and I) and qRT-PCR (J). *n* = 3 for each group.

NF-κB1 (p50) and NF-κB2 (p52) serve as DNA-binding subunits of the NF-κB transcription factor family. Although they lack intrinsic transactivation domains, they regulate gene expression through dimerization with other NF-κB members such as RelA or RelB. As demonstrated in our earlier findings, there was the nuclear translocation of both p50 and p52 induced by coculture, and the mRNA expression of *TRAF1* in AQP2^+^ cells was also regulated by macrophage-derived TRAF2. Therefore, we further investigated whether NF-κB1(p50) and NF-κB2(p52) bind to the predicted sites on the *TRAF1* promoter to promote its transcription. According to the JASPAR prediction, 2 potential *TRAF1* promoter binding sites (PBSs) of NF-κB1 (p50), PBS1 and PBS3, and one NF-κB2 (p52) binding site, PBS2, were identified within the promoter region of the *TRAF1* gene (Fig. 7A). Subsequent chromatin immunoprecipitation (ChIP) combined with qPCR confirmed that PBS2 and PBS3 are potential binding regions within the *TRAF1* promoter (Fig. 7B and C). Dual-luciferase reporter assay further demonstrated that only PBS3 promoted the transcription of *TRAF1* (Fig. 7D to G). Consistently, knockdown of *NF-κB1* decreased both the protein and mRNA level of *TRAF1* (Fig. 7H to J and Fig. S6K). These findings indicated that NF-κB1, a downstream effector of TRAF1 and TRAF2, bound the promoter of *TRAF1* to up-regulate its transcription, which partially explained the increased mRNA of *TRAF1* by *TRAF2.*

### Activation of the NF-κB2 pathway suppresses apoptosis and calcium deposition in AQP2^+^ cells

**Fig. 8.**
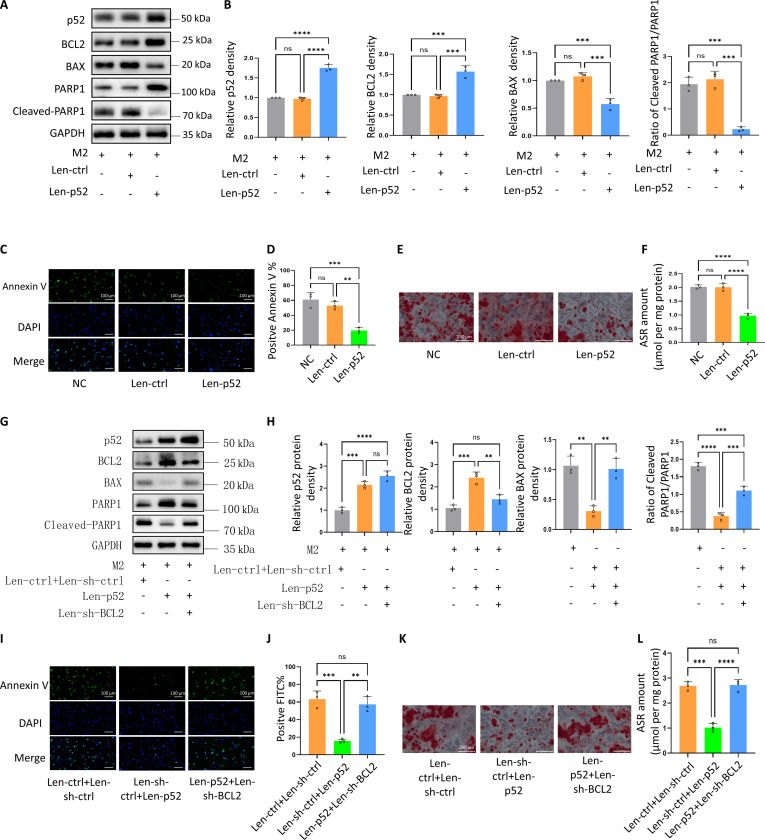
Activation of the NF-κB2 pathway suppresses apoptosis and calcium deposition in AQP2^+^ cells. (A to F) Western blot of BCL2, BAX, and cleaved-PARP1/PARP1 (A and B), TUNEL assay for apoptosis (C and D), and Alizarin Red staining for calcium deposition (E and F) in p52-overexpressing AQP2^+^ cells. *n* = 3 for each group. (G to L) Western blot of BAX and cleaved-PARP1/PARP1 (G and H), TUNEL assay for apoptosis (I and J), and Alizarin Red staining for calcium deposition (K and L) in p52-overexpressing AQP2^+^ cells with Bcl2 knockdown. *n* = 3 for each group.

It was reported that NF-κB2 pathway regulated BCL2 to participate in apoptosis [43]. Here, we found that activation of the NF-κB2 pathway via *p52* overexpression increased BCL2 levels while reducing BAX and cleaved-PARP1/PARP1 in AQP2^+^ cells (Fig. 8A and B), indicating attenuated apoptosis. This anti-apoptotic effect was further supported by fewer TUNEL-positive cells (Fig. 8C and D) and reduced calcium deposition revealed by Alizarin Red staining (Fig. 8E and F). Knockdown of *BCL2* abolished these protective effects, resulting in enhanced apoptosis (Fig. 8G to J and Fig. S6L) and increased calcium deposition (Fig. 8K and L). Together, these findings demonstrated that NF-κB2 activation mitigated calcium deposition in AQP2^+^ cells primarily by suppressing apoptosis.

### Nanocomposite materials enhance M2 exosome-based therapy in *Umod^−/−^* mouse models of kidney stones

**Fig. 9.**
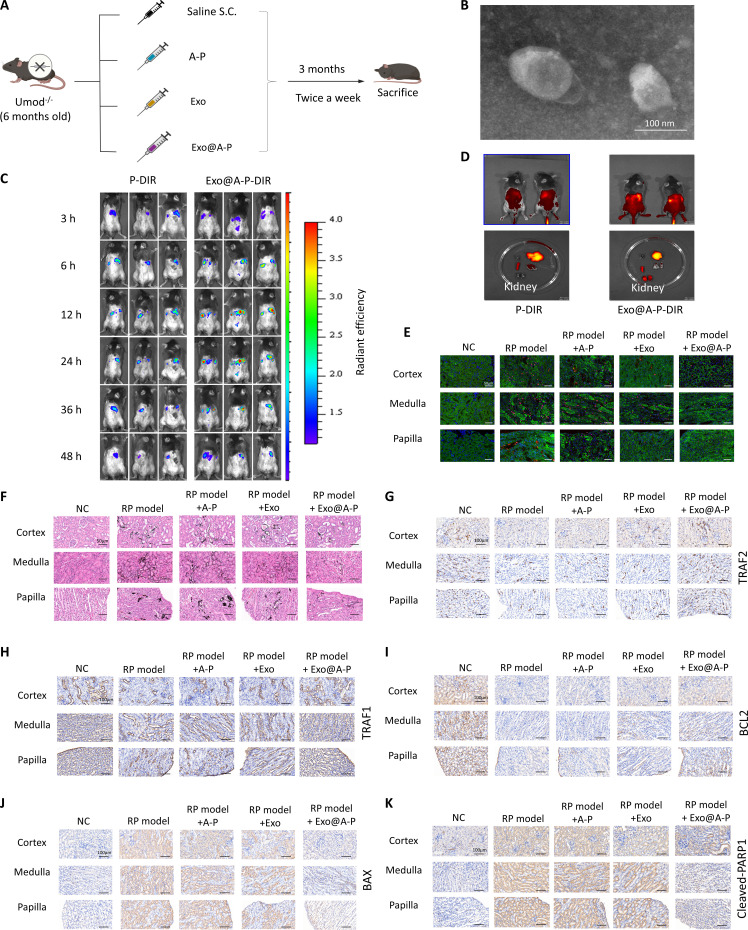
Nanocomposite materials enhance M2 exosome-based therapy in *Umod^−/−^* mouse models of kidney stones. (A) Schematic illustration of the RP model (*Umod^−/−^* mouse) receiving targeted exosome-loaded AQP2^+^ cell membrane-coated PLGA nanoparticle (Exo@A-P) treatment. (B) Transmission electron microscopy (TEM) image of the composite nanomaterial Exo@A-P. (C) In vivo imaging system (IVIS) tracking of fluorescence intensity in various organs of mice across different time points (*n* = 3). (D) Ex vivo fluorescence imaging of major organs harvested 48 h post-injection (*n* = 3). (E) Costaining of AQP2 and TUNEL in mouse kidney across different treatment groups. (F) Von Kossa staining of kidney across different treatment groups. *n* = 6 for each group. (G to K) Immunohistochemical staining of Traf2, Traf1, Bcl2, Bax, and cleaved-Parp1 in mouse kidneys across different treatment groups. *n* = 6 for each group.

To enhance the bioavailability of exosome containing Traf2, AP-Exosome nanoparticle was developed using poly(lactic-co-glycolic acid) (PLGA) encapsulation combined with AQP2^+^ cell membrane for targeted kidney delivery to treat *Umod^−/−^* RP model mice (Fig. 9A and Fig. S7A to C). Transmission electron microscopy (TEM) revealed the morphology of the AQP2^+^ cell membrane-coated exosome-loaded PLGA nanoparticles (Exo@A-P; Fig. 9B), which displayed a clear core–shell architecture, confirming successful nanocomposite fabrication. Dynamic light scattering analysis characterized the physicochemical properties of the nanoparticles. Exo@A-P exhibited a uniform size distribution ranging from 220 to 250 nm with a low polydispersity index (PDI < 0.2) (Fig. S7D and E). Furthermore, the zeta potential measurement showed a moderately positive surface charge, suggesting robust colloidal stability (Fig. S7F). Collectively, these data confirm that Exo@A-P possesses the requisite physicochemical attributes for downstream biological applications.

To evaluate the in vivo biodistribution of the membrane-coated nanoparticles, fluorescently labeled Exo@A-P nanoparticles were administered to mice, and their distribution was tracked using live imaging and ex vivo organ analysis (Fig. 9C to E). In vivo imaging of mice (Fig. 9C) showed that M2 exosome-loaded, AQP2⁺ cell membrane-coated DIR (1,1ʹ-dioctadecyl-3,3,3ʹ,3ʹ-tetramethylindotricarbocyanine iodide)-loaded poly (lactic-co-glycolic acid) nanoparticles (Exo@A-P-DIR) preferentially accumulated in the kidney, with higher fluorescence intensity compared to P-DIR at 48 h post-injection. Ex vivo imaging of organs (Fig. 9D and Fig. S8A) confirmed that AP-DIR exhibited enhanced kidney targeting.

Biochemical analysis indicated that Exo@A-P treatment did not significantly alter serum levels of creatinine, blood urea nitrogen, alanine aminotransferase, or aspartate aminotransferase compared to controls (Fig. S8B), supporting its systemic safety. Furthermore, histopathological examination of 5 major organs (lung, heart, liver, spleen, and kidney) by hematoxylin and eosin (H&E) staining showed preserved tissue architecture without evident inflammatory infiltration or damage (Fig. S8C), further confirming the biosafety profile of Exo@A-P.

Apoptosis of AQP2^+^ cells was significantly increased in RP model mice, which was largely suppressed by exosome treatment or Exo@A-P treatment without influencing urinary calcium excretion (Fig. S9A). Consistently, Von Kossa staining revealed significant calcium deposition in the kidney of RP model mice, and Exo@A-P treatment significantly reduced calcium deposition compared with AQP2^+^ cell membrane-coated PLGA nanoparticle (A-P) treatment or exosome treatment (Fig. 9F and Fig. S9B). Moreover, immunohistochemistry (IHC) staining revealed the up-regulated cleaved-Parp1 and Bax with decreased Bcl2 in RP model mice, which was rescued by Exo@A-P treatment, aligning to the largely increased Traf1 and Traf2 in the Exo@A-P treatment group (Fig. 9G to K and Fig. S9C to G). These results indicated that Exo@A-P attenuated the apoptosis of AQP2^+^ cells and renal interstitial calcium deposition of RP mice.

### The TRAF1–TRAF2/BCL2/BAX/cleaved-PARP1 axis was associated with RP formation

**Fig. 10.**
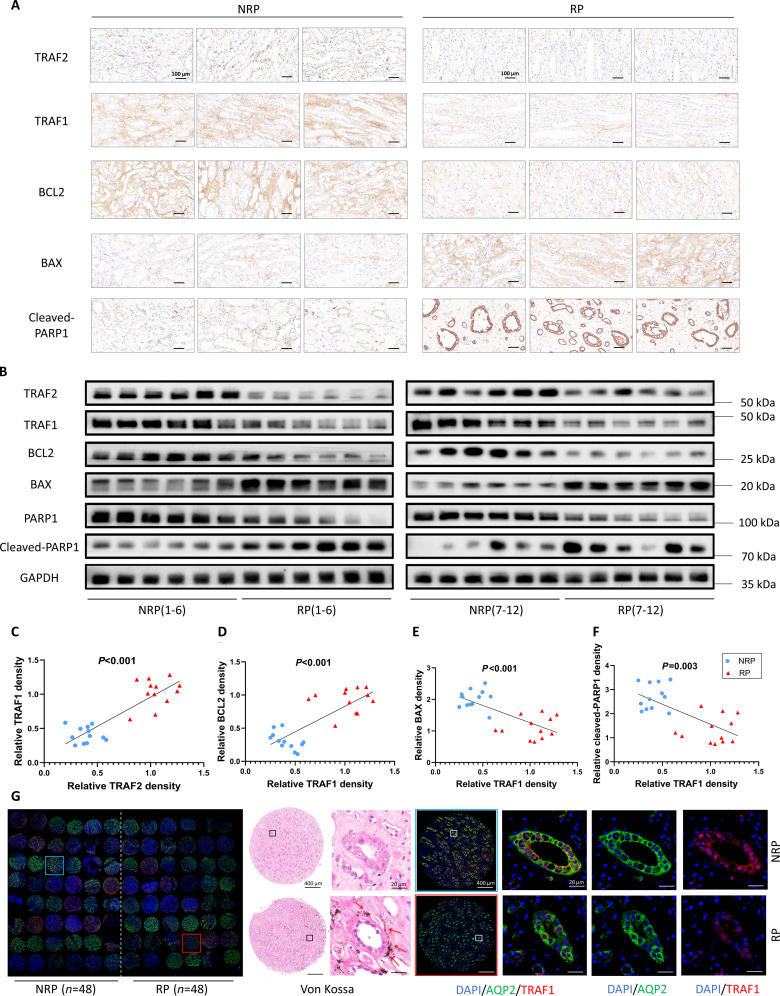
TRAF1, TRAF2, and apoptotic level increase significantly in human RP. (A) Representative immunohistochemical staining of TRAF1, TRAF2, BCL2, BAX, and cleaved-PARP1 in human NRP (*n* = 12) and RP (*n* = 12) tissues. (B) Western blot of TRAF1, TRAF2, BCL2, BAX, PARP1, and cleaved-PARP1 in human NRP (*n* = 12) and RP (*n* = 12) tissues. (C to F) Correlation analysis of TRAF1 and TRAF2, BCL2, BAX, and cleaved-PARP1 identified by Western blot in NRP and RP tissues. (G) TRAF1 and AQP2 were costained in a tissue microarray carrying 48 pairs of NRP and RP tissues, with Von Kossa staining in a serial section.

**Fig. 11.**
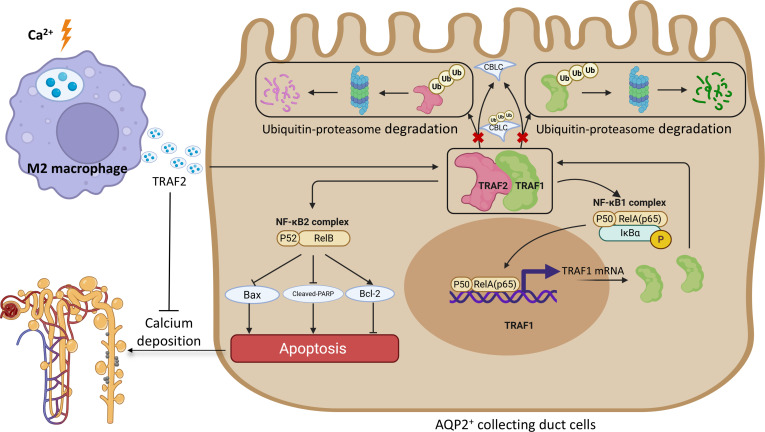
A schematic diagram illustrating that M2 macrophage-derived TRAF2 attenuated the apoptosis of AQP2^+^ collecting duct cells to inhibit RP formation. M2 macrophage-derived exosomal TRAF2 interacted with intracellular TRAF1 to form a stable complex, mutually preventing CBLC-mediated ubiquitination and degradation, and thus activating NF-κB1 and NF-κB2 signaling. NF-κB1 activation enhanced TRAF1 transcription, whereas NF-κB2 activation suppressed apoptosis and subsequent calcium deposition. Therefore, M2 macrophages attenuated the apoptosis of AQP2^+^ collecting duct cells to inhibit RP formation.

Given that our results indicated the key role of TRAF1 and TRAF2 in regulating apoptosis of AQP2^+^ cells to participate in calcium deposition, we further investigated its association with RP formation via IHC staining (Fig. 10A) and Western blot (Fig. 10B) in human RP and NRP tissues. Consistently, TRAF1 and TRAF2 were significantly suppressed in human RP compared with NRP (Fig. S9H and I); BCL2 was significantly decreased, while cleaved-PARP1 and BAX were significantly increased, in human RP (Fig. S9H and I). Moreover, TRAF1 was positively correlated with TRAF2 and BCL2 (Fig. 10C and D), and negatively correlated with BAX and cleaved-PARP1 (Fig. 10E and F). Using a tissue microarray carrying 48 pairs of NRP and RP tissues, we verified that TRAF1 was significantly suppressed in AQP2^+^ cells within RP tissues (Fig. 10G and Fig. S9J). Additionally, TRAF2 was positively associated with BCL2 and negatively associated with BAX and cleaved-PARP1 (Fig. S9K to M). Taken together, our results indicated the protective role of M2-derived exosomes in AQP2^+^ cell apoptosis-induced RP formation via TRAF1–TRAF2/BCL2/BAX/cleaved-PARP1 axis (Fig. 11).

## Discussion

RPs have been traditionally thought to arise from calcium phosphate deposits on the basement membranes of the thin ascending limb of Henle’s loop [15,16], and research on nephrolithiasis has primarily emphasized the loop of Henle or proximal tubular segments [19]. Here, our findings clearly demonstrated that calcium deposits were localized in the interstitium surrounding collecting duct and were closely associated with apoptosis of these cells. Moreover, inhibition of apoptosis markedly reduced calcium accumulation. This observation aligns with previous reports that apoptotic bodies and cellular debris act as nucleation foci for HAP crystallization [28], further supporting our hypothesis that AQP2^+^ cell apoptosis actively drives interstitial calcification during RP initiation.

Several mechanisms may contribute to the lower apoptotic threshold observed in AQP2^+^ cells. First, collecting duct principal cells are continuously exposed to fluctuating luminal ionic and osmotic environments, which may confer distinct calcium-handling properties. Calcium-sensing receptors (CaSRs) and related calcium transport pathways expressed in renal tubular epithelial cells may influence intracellular calcium homeostasis under stress conditions [44]. Second, limited mitochondrial calcium-buffering capacity may render these cells more susceptible to calcium overload, leading to mitochondrial dysfunction and activation of apoptotic pathways [45]. Third, calcium-induced oxidative stress may further exacerbate cellular injury, as collecting duct epithelial cells are particularly responsive to redox imbalance [46]. Collectively, altered calcium sensing, impaired mitochondrial calcium handling, and enhanced oxidative stress may underlie the increased vulnerability of AQP2^+^ cells to calcium-induced apoptosis.

Given that the collecting duct epithelium is a terminal segment exposed to the highest calcium and solute concentrations, it is plausible that apoptotic and oxidative injury in this region establishes a permissive microenvironment for apatite nucleation. Moreover, local alterations in water transport mediated by AQP2 dysregulation may exacerbate calcium supersaturation [47,48], reinforcing the link between collecting duct cells and mineral deposition. In our study, calcium overload induced mitochondrial injury and activation of apoptotic signaling, and treatment with the apoptosis inhibitor Z-VAD-FMK markedly reduced both cell death and calcium deposition. We speculate that membrane components released during apoptosis may provide negatively charged phospholipid surfaces that facilitate calcium phosphate nucleation, similar to the role of matrix vesicles in bone mineralization [49]. Our findings are reinforced by previous studies investigating the cell death of renal tubular epithelial cells, particularly HK-2 cells, in calcium crystallization. Guo et al. observed spatial colocalization of apoptosis, osteogenic trans-differentiation, and calcium deposition in cultured HK-2 cells, suggesting that cell death is a prerequisite for calcification [28]. More recently, Nong et al. [50] reported that pathologic calcium oxalate monohydrate (COM) crystals induce endoplasmic reticulum stress and trigger both apoptosis and pyroptosis in HK-2 cells, leading to calcium deposition. A recent review further emphasized that multiple cell death pathways, including apoptosis, pyroptosis, and ferroptosis, act in concert during different stages of stone formation to promote inflammation and crystal retention [51].

The immune system plays a critical role in the pathogenesis of RP formation and CaOx stones [10]. Macrophages have been reported to phagocytose and destroy CaOx crystals through clathrin-mediated endocytosis while releasing specific inflammatory factors, thereby playing a key role in clearing renal crystal deposits and preventing stone formation [52]. Additionally, it is indicated that pro-inflammatory M1 macrophages promote CaOx crystal formation by up-regulating inflammatory and adhesion factors, while anti-inflammatory M2 macrophages exert protective effects through enhanced phagocytic capacity and suppression of crystal attachment [23,53]. Recent work revealed a novel mechanism by which medullary macrophages directly clear intratubular crystal deposits through integrin β1-dependent transepithelial activity [54]. These studies indicated that macrophages play an important role in calcium deposition and kidney stone formation, but previous research has primarily focused on the direct interactions between macrophages and crystals, mainly focusing on macrophage inflammatory responses and macrophage-mediated crystal phagocytosis [52,55]. In contrast, the role of macrophage interactions with other cell types in RP formation has been less explored.

Here, we found that M2 macrophages exerted a protective role against calcium-induced apoptosis in AQP2^+^ cells. The dynamic interplay between macrophages and collecting duct epithelial cells revealed in this study highlights the importance of immune–epithelial communication in RP pathogenesis. Mechanistically, our data reveal that TRAF2, delivered from M2 macrophages via exosomes, stabilizes TRAF1 in collecting duct cells through mutual protection from ubiquitin-mediated degradation, leading to the activation of both canonical (NF-κB1) and noncanonical (NF-κB2) NF-κB pathways. Activation of these pathways enhanced BCL2 expression, reduced BAX and cleaved-PARP1, and ultimately inhibited apoptosis. These findings are in line with reports that TRAF family members act as crucial mediators of NF-κB signaling in regulating cell survival and immune homeostasis [56]. On the other hand, the inhibitor of apoptosis protein fusion c-IAP2.MALT1 was reported to activate NF-κB signaling independently of TRAF1 and TRAF2, and further studies are warranted to clarify the role of TRAF1/2-mediated NF-κB activation in high calcium-induced apoptosis of AQP2^+^ cells by the application of c-IAP2.MALT1. Nevertheless, the identification of TRAF2 as an exosome-derived effector molecule highlights a novel form of intercellular communication between macrophages and renal epithelial cells in the microenvironment of RP formation.

CBLC is best known for its role in down-regulating receptor tyrosine kinases such as epidermal growth factor receptor (EGFR) [57] by promoting their ubiquitination and endosomal sorting. Our mechanistic dissection reveals that CBLC serves as a critical shared E3 ligase for both TRAF1 and TRAF2. Unlike typical E3–substrate relationships where a single E3 targets multiple unrelated substrates, here we demonstrate that TRAF1 and TRAF2—which form a functional heterodimer—share the same E3 ligase. The formation of the TRAF1–TRAF2 complex occludes the CBLC-binding interface, thereby mutually protecting both proteins from ubiquitin-mediated degradation. This represents a noncanonical, cooperative stabilization mechanism that differs from conventional E3-mediated degradation pathways. In addition, the competitive binding relationship among CBLC, TRAF1, and TRAF2 suggests a dynamic equilibrium wherein the relative abundance of each protein determines the stability of the complex. Our study expands the functional repertoire of CBLC to include the regulation of TRAF family proteins and, more broadly, the control of apoptosis of AQP2^+^ cells and calcification around collecting duct. These findings raise the intriguing possibility that modulating CBLC activity could represent a novel therapeutic approach to stabilize the TRAF1–TRAF2 complex and prevent RP formation.

Membrane coating combined with nanoparticles has emerged as a potential strategy for targeted delivery [58]. Furthermore, exosomes and exosome-mimetic nanocarriers have been shown to possess intrinsic endosomal escape capabilities, thereby enhancing therapeutic efficacy [59]. Based on these findings, our study introduces a translational approach using AQP2^+^ cell membrane-coated PLGA nanoparticles loaded with exosomes to achieve kidney-specific delivery of TRAF2-enriched exosomes. This biomimetic system exploits the natural homing properties of AQP2^+^ membranes to enhance renal tropism and prolong circulation time. Exo@A-P displayed a uniform nanoscale morphology, high encapsulation efficiency, and favorable in vivo biocompatibility, effectively attenuating collecting duct cell apoptosis and calcium deposition in a Umod knockout mouse model of RP. Importantly, no significant off-target toxicity or systemic side effects were observed, underscoring the biosafety of this delivery system. These findings align with emerging evidence that membrane-coated nanocarriers can achieve precise delivery of bioactive molecules to the kidney, overcoming the limitations of conventional systemic therapy [60,61]. In addition, extensive studies have suggested that membrane-coated nanocarriers exhibit favorable biosafety profiles, showing no significant hepatic, cardiac, or immunological toxicity in vivo [62,63]. Their biomimetic surface properties enable immune evasion and reduce nonspecific uptake by major organs, thereby minimizing off-target effects. Direct administration of exosomes often results in rapid clearance and enzymatic degradation, limiting their therapeutic efficacy [64]. Encapsulation within biodegradable PLGA nanoparticles provides structural protection, prolongs circulation time, and enhances the stability and bioactivity of exosomal cargos [65]. The ability to modulate local immune–epithelial signaling suggests that this nanoplatform may provide a potential therapeutic framework for future prevention and treatment strategies in kidney stones. Nevertheless, challenges including large-scale manufacturing, long-term storage stability, batch-to-batch consistency, and regulatory approval remain important barriers for future clinical translation of exosome-based nanotherapeutics.

This study is among the first to focus on the role of AQP2^+^ collecting duct epithelial cells in the initiation of RP, thereby uncovering early calcium deposition adjacent to collecting duct in addition to the ascending limb of Henle’s loop. Multiple subclusters of collecting duct cells have been identified, including intercalated cells, which account for approximately 36% to 40% of epithelial cells in the cortical collecting duct [21], and their specific mechanisms in RP formation are further elucidated. U937-derived macrophages rather than primary human macrophages were employed in this study. Although U937 cells provide a well-established and reproducible model for macrophage-like differentiation and have been widely used to investigate macrophage polarization and exosome biology, they may not fully recapitulate the heterogeneity and functional complexity of primary tissue-resident macrophages in vivo. Additionally, considering the physiological and anatomical differences between mouse and human kidneys, such as the single renal papilla structure in mice versus multiple renal papillae in humans [66], further optimization of the RP mouse model is necessary to strengthen the evidence from our in vivo studies, although *Umod^−/−^* mice represent one of available models to human RP. Furthermore, due to differences in osmotic gradients and ion transport rates between rodent and human kidneys, the validation of RP formation mechanisms in large animals such as pigs and even nonhuman primates represents a future direction that cannot be overlooked. Although no systemic toxicity was observed after 3 months of treatment with Exo@A-P, the long-term safety of repeated administration remains unclear and warrants extended toxicology studies in large animals over 12 months. Additionally, batch variability in exosome loading and membrane coating may affect reproducibility and scalability [67]; therefore, automated manufacturing strategies should be explored to improve consistency. While intravenous delivery is suitable for proof-of-concept, it is suboptimal for long-term prophylaxis, and oral administration remains challenging due to gastrointestinal barriers and enzymatic degradation. Finally, the immunogenicity of AQP2^+^ cell membrane coatings, particularly under repeated dosing, requires further evaluation. Addressing these limitations will be essential for the clinical translation of this biomimetic nanoplatform for preventing RP-associated kidney stones.

In conclusion, AQP2^+^ collecting duct principal cells are identified as a previously underappreciated cellular contributor to interstitial calcium deposition and RP initiation, expanding the current understanding that nephrolithiasis primarily originates from the loop of Henle. We demonstrate that apoptosis of AQP2^+^ epithelial cells actively contribute to mineral nucleation, while M2 macrophage-derived exosomal TRAF2 protects against calcium stress-induced apoptosis by stabilizing TRAF1/TRAF2 complexes and activating NF-κB signaling. Furthermore, our biomimetic Exo@A-P delivery system effectively enhances renal targeting, stabilizes exosomal TRAF2, and mitigates calcium deposition in vivo. Collectively, these findings highlight the collecting duct microenvironment as a key determinant of renal interstitial calcification and reveal a previously unrecognized immune–epithelial crosstalk that governs RP formation. The integration of molecular, cellular, and nanotherapeutic insights offers a new paradigm for understanding and preventing RP-associated kidney stones.

## Materials and Methods

### Clinical sample collection and processing

Renal papilla tissue samples were collected with approval from the Ethics Committee of Xiangya Hospital, Central South University (approval no. 202103089), and written informed consent was obtained from all participants. As described in our previous study [14], the samples were obtained from patients undergoing radical nephrectomy for renal cell carcinoma or ureteral carcinoma at Xiangya Hospital between April 2021 and March 2024. Participants were selected based on the following criteria: preserved renal function, absence or only mild hydronephrosis, no evidence of renal atrophy on computed tomography (CT), and availability of renal papillae located at least 3 cm from the tumor margin. NRPs were defined as renal papillary tissues from patients without a history of urolithiasis and with no detectable calcium deposits on Von Kossa staining. RP samples were defined as renal papillary tissues obtained from patients with CaOx nephrolithiasis or a documented history of CaOx stone formation, in which interstitial calcium deposits were confirmed by histological staining. Five NRP and 4 RP samples from 9 patients were included for scRNA-seq (Table S1). In addition, 26 pairs of NRP and RP samples from 52 patients were used in the subsequent Western blot and histological analysis (Table S11), and 48 pairs of NRP and RP samples from 96 patients were used in tissue microarray (Table S12). To ensure robust comparability and minimize confounding biases, the NRP and RP cohorts were carefully matched based on key demographic and metabolic criteria, including age, gender, body mass index, and baseline comorbidities (hypertension and diabetes).

### scRNA-seq

ScRNA-seq libraries were prepared from fresh renal papilla tissues using the GEXSCOPE Single Cell RNA Library Kit (Singleron) [68]. Briefly, tissues were dissociated into single-cell suspensions using Dissociation Solution (Singleron) incubated for 15 min at 37 °C on the Singleron PythoN Tissue Dissociation System. The suspensions were loaded onto microwell chips with the Singleron Matrix Single Cell Processing System. After collecting the barcoding beads, captured mRNA was reverse-transcribed into cDNA, amplified by PCR, fragmented, and ligated with sequencing adapters. Each library was diluted to 4 nM, pooled, and sequenced on an Illumina NovaSeq 6000 platform [150-base pair (bp) paired-end reads].

Raw reads were processed using the CeleScope pipeline (v1.9.0; https://github.com/singleron-RD/CeleScope) to generate gene expression matrices. Reads were aligned to the GRCh38 reference genome with STAR (v2.6.1a) [69], and unique molecular identifier (UMI)/gene counts per cell were obtained with featureCounts (v2.0.1) [70]. Downstream analysis was performed in Seurat (v4) [68]. After removing low-quality cells [71,72], cells were integrated using SCTransform (glmGamPoi method in Seurat) [73], followed by dimensionality reduction with principal components analysis (PCA), Harmony, and uniform manifold approximation and projection (UMAP) [74]. DEGs were identified with the Seurat FindMarkers function (Wilcox likelihood-ratio test, default parameters), requiring an average log_2_(fold-change) > 0.5. Cell clusters were annotated based on canonical marker expression referenced from the SynEcoSys database and a prior human kidney single-nucleus RNA sequencing (snRNA-seq) study [13]. The details of scRNA-seq analysis were provided in the Supplementary Materials.

### SEM and EDS

As performed in our previous study [14], SEM and EDS were performed on Quanta-200 (FEI, USA). Briefly, 10-μm sections of RP or normal renal papillary tissues were fixed in 2.5% glutaraldehyde and post-fixed in osmium tetroxide. Following dehydration through a graded ethanol series and critical-point drying, samples were sputter-coated with gold. SEM imaging was performed using a Quanta-200 microscope (FEI, USA) at an accelerating voltage of 10 kV. Chemical composition analysis was conducted in parallel using EDS.

### Cell isolation and culture

Primary collecting duct epithelial cells were isolated from normal renal medullary tissue with antibody-coupled magnetic bead separation, as reported by our previous study [14]. Specifically, AQP2^+^ collecting duct cells were extracted using anti-AQP2 immunomagnetic beads. The purity of the isolated cells was confirmed by immunofluorescence, where AQP2^+^ and CD34^−^ cells were identified. These isolated cells were used within 3 to 5 passages for subsequent experiments.

Human lymphoma cells (U937, RRID: CVCL_0007) were obtained from Changsha Abiowell Biotechnology Co. Ltd. (Changsha, China). Both U937 cells and AQP2^+^ collecting duct epithelial cells were cultured in minimum essential medium (MEM; MeilunBio Co. Ltd., Dalian, China) supplemented with 10% fetal bovine serum (FBS; MeilunBio, China) and 1% penicillin–streptomycin (Abiowell Biotechnology Co. Ltd., Hangzhou, China). The cells were maintained at 37 °C in a humidified incubator with 5% CO₂.

For high-calcium induction, a 100 mM calcium chloride dihydrate (CaCl₂·2H₂O) stock solution was prepared in deionized water and serially diluted in complete medium to achieve gradient concentrations of 0, 2.5, 5, 7.5, 10, and 15 mM for subsequent experiments. U937 cells were treated with 100 ng/ml PMA for 8 h to induce adhesion, followed by culture in complete medium for 48 h before further experimentation.

### Cell transfection

As performed in our previous study [14], the expression of target genes was modulated using recombinant lentiviruses. Specifically, recombinant lentiviruses (GenePharma, China) were employed to overexpress *TRAF1* (TNF receptor-associated factor 1), *TRAF2* (TNF receptor-associated factor 2), *BCL2* (B cell lymphoma 2), and *p52*, or to knock down *TRAF1*, *TRAF2*, and *BCL2* via short hairpin RNAs (shRNAs). AQP2^+^ cells were infected with the indicated lentiviruses according to the manufacturer’s protocol. The efficiency of overexpression and knockdown was validated by qRT-PCR and Western blot. All target sequences of recombinant lentiviruses are listed in Table S13.

### Alizarin Red staining and quantification

Cells were fixed with 4% paraformaldehyde for 30 min, stained with Alizarin Red solution for 5 to 10 min, and photographed under an inverted microscope. Bound dye was eluted with 10% acetic acid and neutralized with ammonium hydroxide, and absorbance was measured at 405 nm using a spectrophotometer. Results were normalized to total protein concentration determined by bicinchoninic acid (BCA) assay, as performed in our previous study [14].

### RNA sequencing and differential expression analysis

AQP2^+^ cells were treated with high-calcium medium or cocultured with macrophages for 6 d (*n* = 3 per group). Total RNA was extracted using Trizol reagent (Accurate Biology, China), flash-frozen in liquid nitrogen, and sequenced by Biomarker Technologies Co. Ltd. (Beijing, China) using the Illumina HiSeq X Ten platform. DEGs were identified using the limma R package with thresholds of adjusted *q* < 0.05 and |log₂FC| > 1. KEGG pathway analysis was conducted using the clusterProfiler package. Volcano plots and bar charts were generated for visualization.

### Co-IP

Protein A/G magnetic beads (MedChemExpress, USA) conjugated to antibodies (anti-TRAF1 and anti-TRAF2) were incubated with cell lysates prepared in radioimmunoprecipitation assay (RIPA) buffer (Beyotime, China) containing protease inhibitors overnight at 4 °C. Beads were washed, resuspended in loading buffer, and boiled for 10 min at 98 °C. Supernatants (input and IP) were analyzed by Western blotting.

### ChIP

In accordance with our previous study, ChIP was performed using EpiQuik ChIP Kit (Epigentek, USA) [14]. Briefly, cells were cross-linked with 1% formaldehyde at 37 °C for 10 min, followed by quenching with glycine and cell lysis. The chromatin was then sonicated into fragments ranging from 200 to 1,000 bp and immunoprecipitated with specific antibodies conjugated to protein A/G beads. A positive control (PC) consisted of anti-RNA polymerase II antibody (RPII; included in the kit) and primers targeting the glyceraldehyde-3-phosphate dehydrogenase (GAPDH) promoter region, while normal mouse immunoglobulin G (IgG) (provided in the kit) served as the negative control. The immunoprecipitated DNA was analyzed by qPCR (Table S14), and the resulting qPCR products were further verified by agarose gel electrophoresis.

### Protein–protein interaction structure prediction and interface analysis

The amino acid sequences of target proteins were retrieved from the UniProt database and submitted to the AlphaFold3 online server for protein–protein interaction structure prediction [75], yielding potential models. The model with highest ranking score was selected for interaction interface analysis to identify key interacting amino acid residues. Predicted structures were visualized using the open-source version of PyMOL 2.6 to clearly display the amino acid residues involved in specific protein–protein interactions.

### SPR assay

SPR was used to assess the competitive binding of CBLC to TRAF1 and TRAF2. Following our previous protocols [14], the TRAF1 (T56-30G, SinoBiological, China) working solution was printed onto a 3-dimensional photo-cross-linkable chip (Photo-cross-linker SensorCHIP) using a Biodot AD1520 array printer, with 4 replicate spots per sample and 4 rapamycin positive control spots at the corners. The resulting microarray chip was integrated with a plastic flow channel for sample loading. Recombinant human CBLC protein (1,280 nM, 15659-H20B, SinoBiological, China) was prepared in PBS with 0.1% Tween 20 (pH 7.4) as the running buffer, and 10 mM glycine–HCl (pH 2.0) served as the regeneration buffer. To evaluate CBLC–TRAF1-binding kinetics, the TRAF1 microarray chip was pretreated with TRAF2 solutions (T57-30, SinoBiological, China) at varying concentrations (0, 40, 160, 640, and 2,560 nM) at 0.5 μl/min for 5 min. The CBLC solution was then injected at the same flow rate for 10 min (association), followed by running buffer for 6 min (dissociation). Regeneration was performed with glycine–HCl at 2 μl/min for 200 s. For the TRAF2 microarray chip, the same procedure was applied using TRAF1 solutions for pretreatment, and CBLC–TRAF2 binding kinetics were analyzed under identical conditions. All binding parameters were recorded and processed using the Screen LB 991 Label-free Microarray System (Berthold Technologies, Bad Wildbad, Germany).

### Preparation and characterization of nanoparticles

Based on our previous study [76], PLGA nanoparticles were prepared by nanoprecipitation. PLGA (50 mg; Sigma-Aldrich, St. Louis, MO, USA) was dissolved in dichloromethane (Aladdin, Shanghai, China) and added dropwise into 50 ml of 1% polyvinyl alcohol (Sigma-Aldrich, USA) under 1,000 rpm stirring at 4 °C. After solvent evaporation and washing, PLGA nanoparticles were collected. DIR-loaded poly (lactic-co-glycolic acid) nanoparticles (P-DIR) were obtained by adding DIR tracer (MedChemExpress, Monmouth Junction, NJ, USA). Membrane-coated nanoparticles were formed by co-extrusion of PLGA nanoparticles, AQP2^+^ cell membranes, and macrophage exosomes through a 400-nm membrane using a nano-extruder, followed by purification. Morphology was observed using a transmission electron microscope (Hitachi, Tokyo, Japan).

### Animals

Animal procedures were conducted in strict accordance with institutional guidelines and received ethical approval from the Institutional Experimental Animal Committee of Central South University and Xiangya Hospital (approval no. CSU-2022-0454; XY20240303001). The *Umod^−/−^* mice were used to investigate the therapeutic effects of the nanoparticle system against renal calcium deposition, as it has been reported to be an established and reliable model for RP formation [77,78]. Six-month-old *Umod^−/−^* mice were specifically selected to ensure robust RP model establishment, as younger mice frequently exhibit inconsistent model formation. The *Umod^−/−^* mice (Table S15), carrying deletion of exons 3 to 7 of the *Umod* gene located on mouse chromosome 7 (NCBI Reference Sequence: NM_009470), were purchased from Cyagen Biosciences Co. Ltd. (China).

For the in vivo targeting and retention study, mice were randomly divided into 2 groups and intravenously injected with either P-DIR- or DIR-labeled M2 exosome-loaded, AQP2^+^ cell membrane-coated poly (lactic-co-glycolic acid) nanoparticles (Exo@A-P-DIR). Fluorescence distribution was monitored using in vivo imaging system (IVIS) at 3, 6, 12, 24, 36, and 48 h post-injection, followed by ex vivo fluorescence analysis of major organs (heart, liver, spleen, lung, and kidney) at the 48-h endpoint.

For the long-term therapeutic efficacy study, mice were randomly assigned to 5 groups: a wild-type normal control (NC) group, a *Umod^−/−^* model group receiving saline, and 3 *Umod^−/−^* treatment groups receiving AQP2^+^ cell membrane-coated PLGA nanoparticles (RP model + A-P), free exosomes (RP model + Exo), or exosome-loaded AQP2^+^ cell membrane-coated PLGA nanoparticles (RP model + Exo@A-P). Treatments were administered twice a week for a duration of 3 months. Upon completion of the treatment regimen, all mice were euthanized, and kidney tissues were immediately harvested. The renal tissues were fixed in 4% paraformaldehyde, embedded in paraffin, and sectioned for comprehensive histological examination. The staining protocols included H&E staining for morphological assessment, Von Kossa staining for the specific detection and quantification of calcium deposits, and IHC staining to evaluate the expression of therapeutic targets and apoptotic markers.

### Statistical analysis

All statistical analyses were performed using GraphPad Prism 10 (GraphPad Software, San Diego, CA, USA) and R software (version 4.4.2). Data distribution normality was assessed using the Shapiro–Wilk test prior to parametric analysis. For comparisons between 2 groups, Student’s *t* test (2-tailed, unpaired) was applied for normally distributed data, while the Mann–Whitney *U* test was used for non-normally distributed data. For comparisons among multiple groups, one-way analysis of variance (ANOVA) was performed as appropriate, followed by Tukey’s post hoc test for multiple comparisons. Homogeneity of variance was evaluated using Levene’s test. For categorical variables, the chi-square test or Fisher’s exact test was used as appropriate. Correlation analyses were performed using Pearson’s correlation coefficient for normally distributed data or Spearman’s rank correlation for nonparametric data.

Data are presented as mean ± standard deviation (SD) or median with interquartile range (IQR), depending on data distribution. The number of biological and technical replicates (*n*) is indicated in each figure legend. To aid readability, *P* values are shown as exact values where possible; otherwise, significance levels are indicated as *P < 0.05, **P < 0.01, ***P < 0.001. A 2-sided *P* value of <0.05 was considered statistically significant.

## Ethical Approval

All patients gave written informed consent for collection of clinical renal tissue specimens and information. All animal experiments were in compliance with the European Community Guidelines for the use of experimental animals. Ethics approval was granted by the Xiangya Hospital Ethics Committee (approval no. 202103089) and the Institutional Experimental Animal Committee of Xiangya Hospital (approval no. CSU-2022-0454; XY20240303001).

## Data Availability

The single-cell RNA sequencing generated in this study have been deposited in the NCBI Sequence Read Archive (SRA) database under BioProject accession numbers PRJNA1380965 and PRJNA1380926.
