# Supplementary material for: M2 Macrophages Attenuate AQP2+ Collecting Duct Cell Apoptosis via the TRAF1–TRAF2 Complex to Suppress Randall’s Plaque Formation
Source: Research (Wash D C). 2026 Jul 16;9:1364. doi: 10.34133/research.1364 (PMC13373320; doi:10.34133/research.1364)
Supplement: Supplementary 1 — Supplementary Methods Figs. S1 to S9 Tables S1 to S17 [file research.1364.f1.zip › Supplementary Materials-submitted-R3.docx]

**Supplementary Materials for “****M2 Macrophages Attenuate AQP2⁺ Collecting Duct Cell Apoptosis via the TRAF1–TRAF2 Complex to Suppress Randall’s Plaque Formation”**

**1. Supplementary Methods**

**1.1 RNA extraction and quantitative real-time PCR (qRT-PCR)**

Total RNA was extracted using the SteadyPure Rapid RNA Extraction Kit (Accurate Biology, China). RNA purity and concentration were assessed using a NanoDrop spectrophotometer (Thermo Fisher, USA). Complementary DNA (cDNA) was synthesized using the Evo M-MLV Reverse Transcription Premix Kit (Accurate Biology, China). Quantitative PCR was performed with SYBR Green Pro Taq HS Premix qPCR Kit II (Accurate Biology, China) on a Mastercycler® RealPlex system (Eppendorf, Germany). GAPDH was used as an internal control, and relative gene expression was calculated using the 2⁻ΔΔCT method. Primers used were listed in the Table S16.

**1.2 Western Blot Analysis**

Protein samples were separated using 10–12% SDS-PAGE and transferred to PVDF membranes (Millipore, USA). After blocking with rapid blocking buffer (Epizyme, China) for 15 min, membranes were incubated overnight at 4°C with specific primary antibodies and then with HRP-conjugated secondary antibodies (1:5000 dilution) for 1 h at room temperature. Immunoreactive bands were detected using an ECL substrate (ZenBio, China) and visualized with an Amersham ImageQuant imaging system (Cytiva, UK). Band intensity was quantified using ImageJ software. The detailed information about antibodies was also listed in Table S17.

**1.3 Immunofluorescence Staining**

Cells were rinsed with phosphate-buffered saline (PBS), fixed with 4% paraformaldehyde at room temperature for 10-15 min, and blocked with 5% goat serum (Solarbio Life Sciences, Beijing, China) for 30 min. Primary antibodies were applied overnight at 4°C according to the manufacturer’s instructions, followed by incubation with fluorescently labeled secondary antibodies (1:400 dilution) for 1 h in the dark. Nuclei were counterstained with DAPI (Abiowell, China) for 5 min. Fluorescence imaging was performed using a confocal fluorescence microscope (Leica STELLARIS 8 STED, German). The detailed information about the primary and secondary antibodies was provided in the Table S17.

**1.4** **Histological and Immunohistochemical Staining**

As performed in our previous study[1], paraffin sections were baked at 60°C, deparaffinized, and rehydrated through graded ethanol. Von Kossa staining (Solarbio, China) was used to detect calcium deposits, and H&E staining (Solarbio, China) for morphology. For immunohistochemistry, sections underwent antigen retrieval, blocking with 5% goat serum, incubation with primary and HRP-conjugated secondary antibodies, and visualization with DAB substrate. Images were captured using a light microscope (Leica Microsystems, Germany) and analyzed with ImageJ. As performed in a previous study[2], the IHC Toolbox plugin in ImageJ was used to quantitatively analyze images of DAB staining[3]. The color threshold for the average grey value was set between 0 and 254. A value of 0 corresponded to black, dark-stained areas, while 254 corresponded to white, unstained areas, resulting in an inverse correlation between DAB staining intensity and the average grey value.

**1.5 scRNA-seq analysis**

Computational analysis was conducted using Scanpy (v1.9.3) in Python 3.10[4]. For each sample, the expression matrix was filtered as follows: (1) cells with fewer than 200 genes or within the top 2% of gene counts were excluded; (2) cells within the top 2% of UMI counts were removed; (3) cells with >50% mitochondrial content were filtered out; (4) genes detected in fewer than 5 cells were omitted. The raw count matrix was normalized by total counts per cell and log-transformed. The top 2,000 variable genes were identified using the ‘seurat_v3’ flavor. Batch effects across samples were corrected with harmonypy (v0.0.6) based on the first 50 principal components (PCs)[5], using theta=2 and sigma=0.1. The resulting 27 Harmony‑adjusted components were used for downstream clustering and dimensionality reduction. Cell clustering was performed with the Louvain algorithm (resolution=1.2) and visualized via UMAP. For cluster annotation, marker genes were selected from cluster‑specific differentially expressed genes (DEGs) and cross‑referenced with the SynEcoSys database, requiring ≥3 concordant markers per cluster. Annotation confidence was assessed by Jaccard similarity (>0.4) and a random forest classifier (>0.7); all major clusters achieved >0.85.

**1.6** **Exosome isolation**

Cells were washed 3 times with PBS, and cultured for 48 hours using the complete medium without exosomes (FBS was centrifuged at 100,000 × g, 4 ℃). Cell culture supernatant was sequentially centrifuged at 300 × g, 4 ℃ for 10min, 2000 × g, 4 ℃ for 20min, 10,000 × g, 4 ℃ for 30 min. Supernatant was transferred into a new ultracentrifuge tube, and centrifuged at 100,000×g, 4 ℃ for 90 min. Precipitate was resuspended in 50 mL PBS, centrifuged at 100,000 × g, 4 ℃ for 90 min, and supernatant was discarded. The precipitate was resuspended in 200 μL precooled PBS and stored at -80 ℃.

**1.7 TUNEL Staining**

Cells were fixed with 4% paraformaldehyde, permeabilized with PBS containing 3% Triton X-100 for 5 min, and stained using a One-Step TUNEL Apoptosis Detection Kit (Beyotime, Shanghai, China) following the manufacturer’s instructions. Fluorescence images were obtained with an inverted fluorescence microscope.

**1.8 Flow Cytometric Analysis of Apoptosis**

Cells were digested with trypsin without EDTA, washed twice with PBS, and resuspended in 100 μL of 1× Binding Buffer. Annexin V-FITC and propidium iodide (PI) were added (5 μL each; Beyotime, China), followed by 10–15 min dark incubation at room temperature. Samples were analyzed within 1 h using a flow cytometer (BD Biosciences, USA).

**1.9 Agarose Gel Electrophoresis**

Agarose gels (2%) were prepared in 1 × TAE buffer containing SYBR Safe DNA Gel Stain (Thermo Fisher Scientific, USA). DNA samples were loaded alongside a DNA ladder and electrophoresed at 150 V for 30–40 min. Bands were visualized using a Gel Doc imaging system (Bio-Rad, Hercules, CA, USA).

**2. Supplementary Tables**

**Table S1.** Clinical characteristics of patients included in the scRNA-Seq analysis.

| **Characteristics** | **NRP group**  **(n=5)** | **RP group**  **(n=4)** | ***P* value** |
| --- | --- | --- | --- |
| Age, years, mean (SD) | 53.8 (7.2) | 52.0 (7.8) | 0.75 |
| Gender, female/male | 1/4 | 1/3 | >0.99 |
| BMI, kg/m^2^, mean (SD) | 24.0 (5.0) | 25.1 (5.3) | 0.76 |
| Comorbidities, n (%) |  |  |  |
| Hypertension | 1 (20.0%) | 1 (25.0%) | >0.99 |
| Diabetes mellitus | 0 (0) | 0 (0) | >0.99 |
| Blood pressure medication, n (%) | 1 (20.0%) | 1 (25.0%) | >0.99 |
| Insulin, n (%) | 0 (0) | 0 (0) | >0.99 |
| Tumor size, cm, mean (SD) | 4.3 (0.8) | 4.6 (0.9) | 0.65 |
| Hydronephrosis degree (none/mild) * | 4/1 | 3/1 | >0.99 |
| Stone recurrence history, n (%) | 0 (0) | 0 (0) | >0.99 |

All NRP and RP samples from patients undergoing Radical Nephrectomy (RN) for tumors. *The Society of Fetal Urology grading system was used to determine the hydronephrosis degree, and those with severe hydronephrosis were routinely excluded in the current study. SD, standard deviation. NRP, normal renal papillae; RP, renal papillae with Randall’s Plaques.

**Table S2 (excel file). Differentially Expressed Genes (DEGs) of collecting duct cells between normal renal papilla (NRP) and Randall’s Plaque (RP) tissues identified by scRNA-Seq.**

**Table S3 (excel file). Differentially Expressed Genes (DEGs) of AQP2^+^ cells co-culture with or without M2 identified by bulk RNA-Seq.**

**Table S4 (excel file). Proteins of exosomes identified by MS from the supernatant of AQP2^+^ cells.**

**Table S5 (excel file). Proteins of exosomes identified by MS from the supernatant of AQP2^+^ cells co-cultured with M2 macrophages.**

**Table S6 (excel file). Proteins binding to TRAF1 identified by CO-IP and MS from AQP2^+^ cells.**

**Table S7 (excel file). Proteins binding to TRAF1 identified by CO-IP and MS from AQP2^+^ cells co-cultured with M2 macrophages.**

**Table S8 (excel file). E3 ligases of TRAF1 predicted by Ubibrowser.**

**Table S9 (excel file). E3 ligases of TRAF2 predicted by Ubibrowser.**

**Table S10.** Amino acid sequences of TRAF1 and TRAF2 mutants

|  | Amino acid sequences |
| --- | --- |
| TRAF1-mut | MASSSGSSPRPAPDENEFPFGCPPTVCQDPKEPRALCCAGCLSENPRNGEDQICPKCRGEDLQSISPGSRLRTQEKAHPEVAEAGIGCPFAGVGCSFKGSPQSVQEHEVTSQTSHLNLLLGFMKQWKARLGCGLESGPMALEQNLSDLQLQAAVEVAGDLEVDCYRAPCSESQEELALQHFMKEKLLAELEGKLRaaEaaaAaLaKEVEASaLALATSaHQSQLDRERILSLEQRVVELQQTLAQKDQALGKLEQSLRLMEEASFDGTFLWKITNVTRRCHESACGRTVSLFSPAFYTAKYGYKLCLRLYLNGDGTGKRTHLSLFIVIMRGEYDALLPWPFRNKVTFMLLDQNNREHAIDAFRPDLSSASFQRPQSETNVASGCPLFFPLSKLQSPKHAYVKDDTMFLKCIVETST |
| TRAF2-mut | MAAASVTPPGSLELLQPGFSKTLLGTKLEAKYLCSACRNVLRRPFQAQCGHRYCSFCLASILSSGPQNCAACVHEGIYEEGISILESSSAFPDNAARREVESLPAVCPSDGCTWKGTLKEYESCHEGRCPLMLTECPACKGLVRLGEKERHLEHECPERSLSCRHCRAPCCGADVKAHHEVCPKFPLTCDGCGKKKIPREKFQDHVKTCGKCRVPCRFHAIGCLETVEGEKQQEHEVQWLREHLAMLLSSVLEAKPLLGDQSHAGSELLQRCESLEKaaATFaNaaCVLaRaaERVAMTAEAaSRQHRLDQDKIEALSSKVQQLERSIGLKDLAMADLEQKVLEMEASTYDGVFIWKISDFARKRQEAVAGRIPAIFSPAFYTSRYGYKMCLRIYLNGDGTGRGTHLSLFFVVMKGPNDALLRWPFNQKVTLMLLDQNNREHVIDAFRPDVTSSSFQRPVNDMNIASGCPLFCPVSKMEAKNSYVRDDAIFIKAIVDLTGL |

Mutation of TRAF1 (TRAF1-mut): V196A; F197A; N199A; I200A; V201A; V203A; N205A; H212A; I219A. Mutation of TRAF2 (TRAF2-mut): K278A; T279A; E283A; I285A; V286A; N290A; E292A; V293A; C303A

**Table S11.** Clinical characteristics of patients included in WB and histological analysis.

| Characteristics | NRP group  (n=26) | RP group (n=26) | *P* value |
| --- | --- | --- | --- |
| Age (years), mean±SD (range) | 54.5±4.3  (40-66) | 56.3±5.8  (39-62) | 0.21 |
| Gender (M/F) | 14/12 | 13/13 | 0.78 |
| Urinary calcium/creatinine,  mean±SD (range) | 0.162 ± 0.058, (0.113, 0.224) | 0.225±0.071,  (0.121, 0.314) | <0.05 |
| Hydronephrosis degree (none/mild)* | 19/7 | 16/10 | 0.37 |
| Comorbidities, n (%) |  |  |  |
| Hypertension | 7 (26.9%) | 6 (23.1%) | 0.75 |
| Diabetes mellitus | 4 (15.4%) | 3 (11.5%) | >0.99 |
| Blood pressure medication, n (%) | 5 (19.2%) | 5 (19.2%) | >0.99 |
| Insulin, n (%) | 2 (7.7%) | 1 (3.8%) | >0.99 |
| Tumor size (cm), mean±SD (range) | 5.6±1.4  (4.5-6.7) | 5.3±2.1  (4.0-7.5) | 0.53 |
| Stone recurrence history, n (%) | 0 (0) | 6 (23.1%) | 0.02 |

*The Society of Fetal Urology grading system was used to determine the hydronephrosis degree, and those with severe hydronephrosis were routinely excluded in the current study. M, male; F, female; SD, standard deviation. NRP, normal renal papillae; RP, renal papillae with Randall’s plaques.

**Table S12**. Clinical characteristics of patients included in tissue microarray.

| Characteristics | NRP group  (n=48) | RP group (n=48) | P value |
| --- | --- | --- | --- |
| Age (years), mean±SD (range) | 53.1±3.7  (42-67) | 54.0±4.1  (41-65) | 0.26 |
| Gender (M/F) | 28/20 | 26/22 | 0.67 |
| Urinary calcium/creatinine,  mean±SD (range) | 0.158±0.045  (0.105, 0.210) | 0.231±0.068  (0.118, 0.320) | <0.01 |
| Hydronephrosis degree (none/mild)* | 35/13 | 30/18 | 0.27 |
| Comorbidities, n (%) |  |  |  |
| Hypertension | 13 (27.1%) | 11 (22.9%) | 0.63 |
| Diabetes mellitus | 7 (14.6%) | 6 (12.5%) | 0.76 |
| Blood pressure medication, n (%) | 8 (16.7%) | 7 (14.6%) | 0.78 |
| Insulin, n (%) | 4 (8.3%) | 2 (4.2%) | 0.68 |
| Tumor size (cm), mean±SD (range) | 5.4±1.5  (4.2-6.8) | 5.2±1.8  (3.9-7.4) | 0.55 |
| Stone recurrence history, n (%) | 0 (0) | 9 (18.8%) | <0.01 |

*The Society of Fetal Urology grading system was used to determine the hydronephrosis degree, and those with severe hydronephrosis were routinely excluded in the current study. M, male; F, female; SD, standard deviation. NRP, normal renal papillae; RP, renal papillae with Randall’s plaques.

**Table S13.** Targeted sequences of recombinant lentiviruses.

| Targeted Gene | Reconstituted lentivirus | Targeted sequences (5’-3’) |
| --- | --- | --- |
| *TRAF1* | Len-sh1-*TRAF1* | CATTGTGGAGACCAGCACTTA |
|  | Len-sh2-*TRAF1* | TGTCGCTCTTCATCGTGATCA |
|  | Len-sh3-*TRAF1* | GCAGTCTCAATGGGTCAGAAA |
| *TRAF2* | Len-sh1-*TRAF2* | CCCTTGCAGATTCCACGCCAT |
|  | Len-sh2-*TRAF2* | CGAGACGGTAGAGGGTGAGAA |
|  | Len-sh3-*TRAF2* | GTGTTCACGAGGGCATATATG |
| *BCL2* | Len-sh1-*BCL2* | ATGGTTATCTTACGACTGTTA |
|  | Len-sh2-*BCL2* | AGCCGAAGACCACCCACGAAT |
|  | Len-sh3-*BCL2* | TGGATGACTGAGTACCTGAAC |
| NF-κB1 | Len-sh1- *NF-κB1* | CGCCTGAATCATTCTCGATTT |
|  | Len-sh2- *NF-κB1* | CCAGAGTTTACATCTGATGAT |
|  | Len-sh3- *NF-κB1* | GCCTGAACAAATGTTTCATTT |
| CBLC | Len-sh1- CBLC | CCCAGAAATGCCCAGCCGAAA |
|  | Len-sh2- CBLC | GAAACCGAAATAAACTGCCAA |
|  | Len-sh3- CBLC | CGTGTCCATCTTCGAGTTCGA |

**Table S14. The primers used in qPCR for the products of chromatin immunoprecipitation (ChIP).**

| **Gene** | **Primers sequences (5’-3’)** |
| --- | --- |
| *TRAF1* (Promoter binding site 1) | F: 5’-CTAGATGGAGTCTCGCACTC-3’  R: 5’-CCTCTGTGGGGCCATTCAAT-3’ |
| *TRAF1* (Promoter binding site 2) | F: 5’-GGGAAACTGAGTCTGAGGGC-3’  R: 5’-AACCCAATAAGCTTGGGCGA-3’ |
| *TRAF1* (Promoter binding site 3) | F: 5’-TGCCTTTTCCTGGGTGTTGT-3’  R: 5’-TAGGCACCAAGCAATCCCTG-3’ |

**Table S15. Primer sequences designed for mouse genotyping.**

| **Gene** | **Forward primer 5′–3′** | **Reverse primer 5′–3′** |
| --- | --- | --- |
| *Umod* (WT: 529bp) | GTGATGTTTCTGGTGGGACAAGG | ATTCAGAACACCGTCCTGCG |
| *Umod* (KO: 458bp) | GTGATGTTTCTGGTGGGACAAGG | CAGAGGGTGGAAATTGGGTGAAC |

**Table S16. The primers for qRT-PCR.**

| **Gene** | **Primers sequences (5’-3’)** |
| --- | --- |
| *BIRC3* | F: 5’- AAGCTACCTCTCAGCCTACTTT-3’  R: 5’- CCACTGTTTTCTGTACCCGGA-3’ |
| *GADD45B* | F: 5’- TACGAGTCGGCCAAGTTGATG-3’  R: 5’- GGATGAGCGTGAAGTGGATTT-3’ |
| *NFKBIA* | F: 5’- CTCCGAGACTTTCGAGGAAATAC-3’  R: 5’- GCCATTGTAGTTGGTAGCCTTCA-3’ |
| *DDIT3* | F: 5’- GGAAACAGAGTGGTCATTCCC-3’  R: 5’- CTGCTTGAGCCGTTCATTCTC-3’ |
| *FOS* | F: 5’- ATGGACCAGTGAAGCGATCAT-3’  R: 5’- GTTCCTCCAAACTAGAAGCAGC-3’ |
| *IL3RA* | F: 5’- AGACGCCGACTATTCTATGCC-3’  R: 5’- CGGTGTAGTTGGTCACTTCACA-3’ |
| *BCL2* | F: 5’- TTGCCAGCCGGAACCTATG-3’  R: 5’- CGAAGGCGACCAGCAATGATA-3’ |
| *GADD45G* | F: 5’-GCACGCAGCCTACTAGGTG-3’  R: 5’-CGAACTGCTTAGCCGCGTA-3’ |
| *BCL2A1* | F: 5’- TACAGGCTGGCTCAGGACTAT-3’  R: 5’- CGCAACATTTTGTAGCACTCTG-3’ |
| *TRAF2* | F: 5’-TCCCTGGAGTTGCTACAGC-3’  R: 5’-AGGCGGAGCACAGGTACTT-3’ |
| *TRAF1* | F: 5’-TCCTGTGGAAGATCACCAATGT-3’  R: 5’-GCAGGCACAACTTGTAGCC-3’ |

**Table S17.** The details of antibodies used in the current study.

| Antibody | Host | Company Cat No. |
| --- | --- | --- |
| Anti-β-tubulin | Mouse | 66240-1-IgProteintech, China |
| Anti-GAPDH | Rabbit | 10494-1-AP, Proteintech, China |
| Anti-β-Actin | Mouse | 66009-1-Ig, Proteintech, China |
| Anti- BCL2 | Mouse | ab182858, Abcam, UK |
| Anti-BAX | Mouse | ab32503, Abcam, UK |
| Anti-Arg-1 | Mouse | 66129-1-lg, Proteintech, China |
| Anti-Cleaved PARP1 | Rabbit | ab32064, Abcam, UK |
| Anti-PARP1 | Rabbit | ab191217, Abcam, UK |
| Anti-Caspase3 | Rabbit | 19677-1-AP, Proteintech, China |
| Anti-iNOS | Rabbit | 18985-1-AP, Proteintech, China |
| Anti-IL-1β | Mouse | 66737-1-Ig, Proteintech, China |
| Anti- IL-10 | Mouse | 60269-1-Ig, Proteintech, China |
| Anti-TRAF1 | Rabbit | ab300075, Abcam, UK |
| Anti-TRAF2 | Rabbit | ab126758, Abcam, UK |
| Anti-CD63 | Mouse | 67605-1-Ig, Proteintech, China |
| Anti-CD9 | Mouse | 60232-1-Ig, Proteintech, China |
| Anti-Ubiquitin | Rabbit | 10201-2-AP, Proteintech, China |
| Anti-IκBα | Rabbit | 10268-1-AP, Proteintech, China |
| Anti-p-IκBα | Rabbit | 82349-1-RR, Proteintech, China |
| Anti-IKKβ | Rabbit | ab32135, Abcam, UK |
| Anti-p-IKKβ | Rabbit | ab194528, Abcam, UK |
| Anti-IKKα | Rabbit | ab32041, Abcam, UK |
| Anti-p-IKKα | Rabbit | ab17943, Abcam, UK |
| Anti-NF-κB1 | Rabbit | 14220-1-AP, Proteintech, China |
| Anti-NF-κB2 | Rabbit | 15503-1-AP, Proteintech, China |
| Anti-LaminA/C | Rabbit | 10298-1-AP, Proteintech, China |
| Anti-Runx2 | Mouse | ab76956, Abcam, UK |
| Anti-OCN | Rabbit | ab133612, Abcam, UK |
| Anti-BMP2 | Mouse | 66383-1-Ig, Proteintech, China |
| Anti-OMD | Rabbit | ab154249, Abcam, UK |
| HRP-conjugated anti-Rabbit IgG | Goat | SA00001-2, Proteintech, China |
| HRP-conjugated anti-Mouse IgG | Goat | SA00001-1, Proteintech, China |
| Anti- Rabbit IgG H&L (Alexa Fluor®488) | Goat | ab150077, Abcam, UK |
| Anti-Rabbit IgG H&L (Alexa Fluor®594) | Goat | ab150080, Abcam, UK |
| Anti-Mouse IgG H&L (Alexa Fluor®594) | Goat | ab150116, Abcam, UK |
| Anti-Cbl-c | Rabbit | ab34750, Abcam, UK |
| HA tag Polyclonal antibody | Rabbit | 51064-2-AP, Proteintech, China |
| DYKDDDDK tag Monoclonal antibody (Binds to FLAG® tag epitope) | Mouse | 66008-4-Ig, Proteintech, China |

**Supplementary Figures**


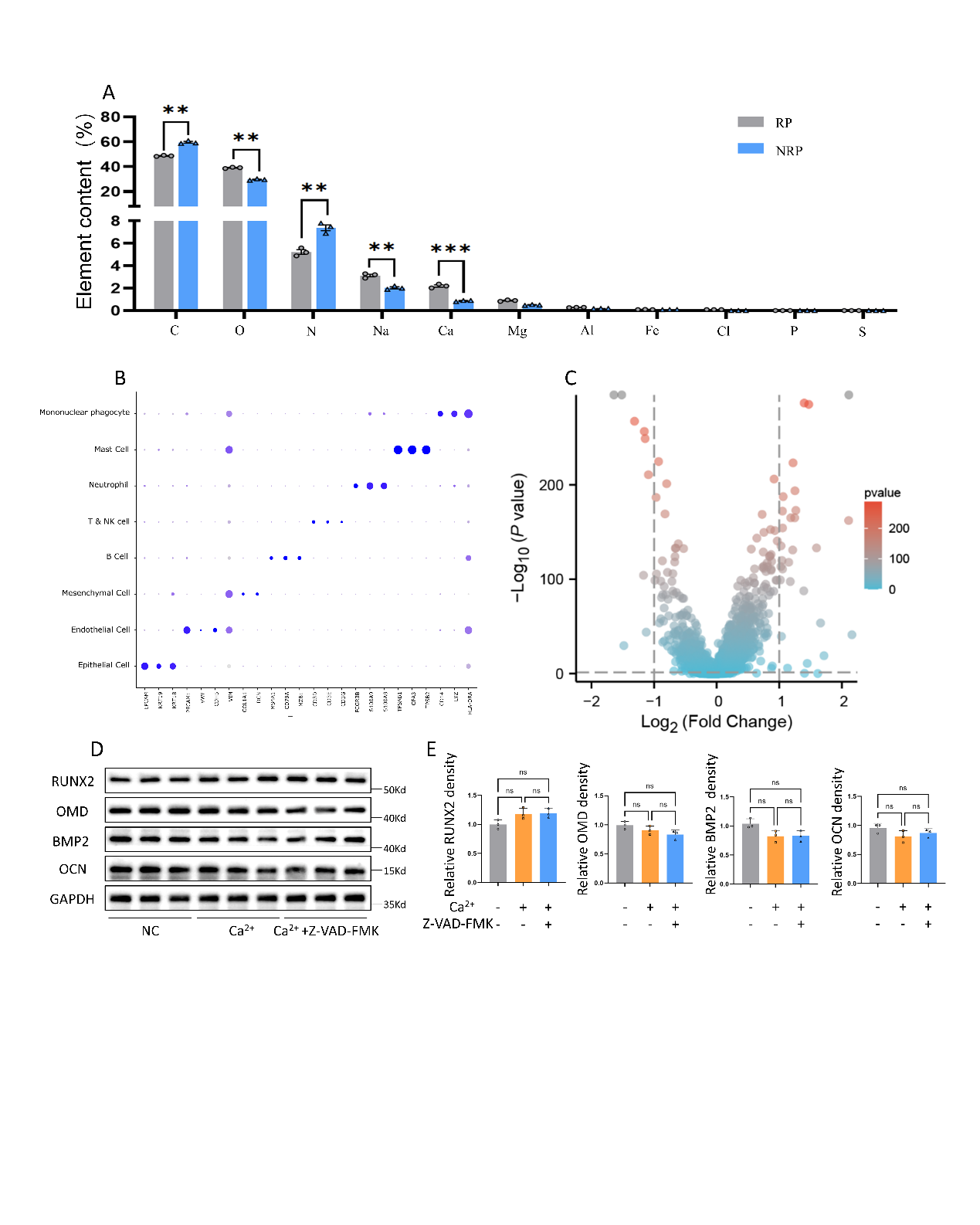


**Figure S1.**

**(A)** Comparison of element component identified by Energy dispersive spectroscopy (EDS) elemental analysis of NRP (n=3) and RP (n=3) tissues.

**(B)** Dot plot of highly variable genes across cell populations of single-cell transcriptomes from NRP (n=3) and RP (n=3) tissues.

**(C)** Volcano plot of differentially expressed genes in AQP2⁺ cells from NRP and RP tissues.

**(D-E)** Western blot analysis of osteogenic-related protein expression (RUNX2; BMP2; OMD; OCN) in AQP2⁺ cells under high-calcium stimulation (5mM; 6 days) with or without Z-VAD-FMK treatment (20 μM; n=3).


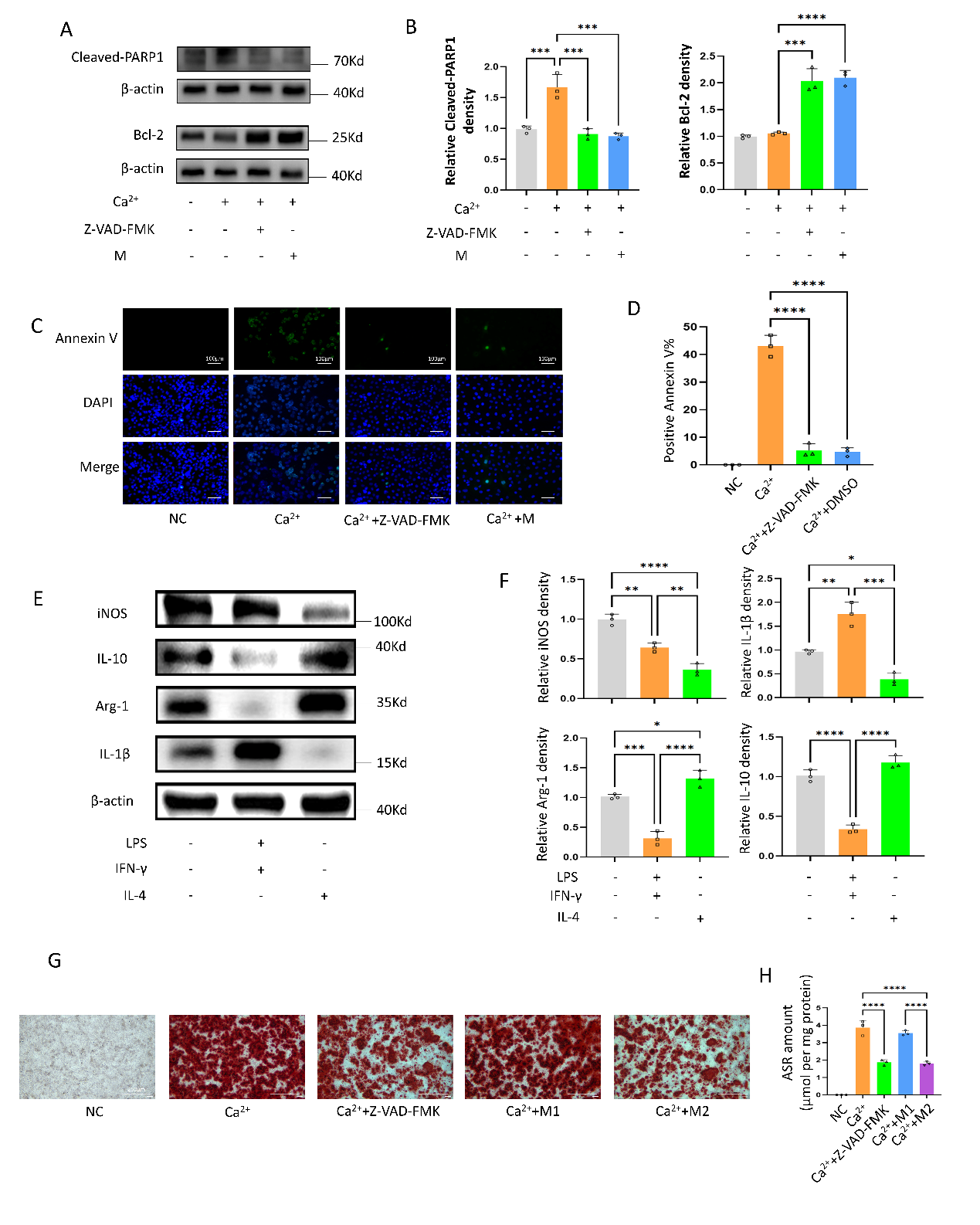


**Figure S2.**

**(A-B)** Western blot of cleaved-PARP1 and Bcl-2 in AQP2⁺ cells treated with Z-VAD-FMK (20 μM; n=3) or co-cultured with M2 macrophages (n=3).

**(C-D)** TUNEL staining of AQP2⁺ cells treated with Z-VAD-FMK (20 μM; n=3) or co-cultured with M2 macrophages (n=3).

**(E-F)** PMA-induced U937 macrophages were polarized with LPS+IFN-γ (M1) or IL-4 (M2) for 24 h, and iNOS, IL-1β, Arg-1 and IL-10 were examined by Western blot (n=3).

**(G-H)** Alizarin Red staining to assess the effects of M1 or M2 macrophages co-culture on calcium salt deposition in AQP2⁺ cells (n=3).


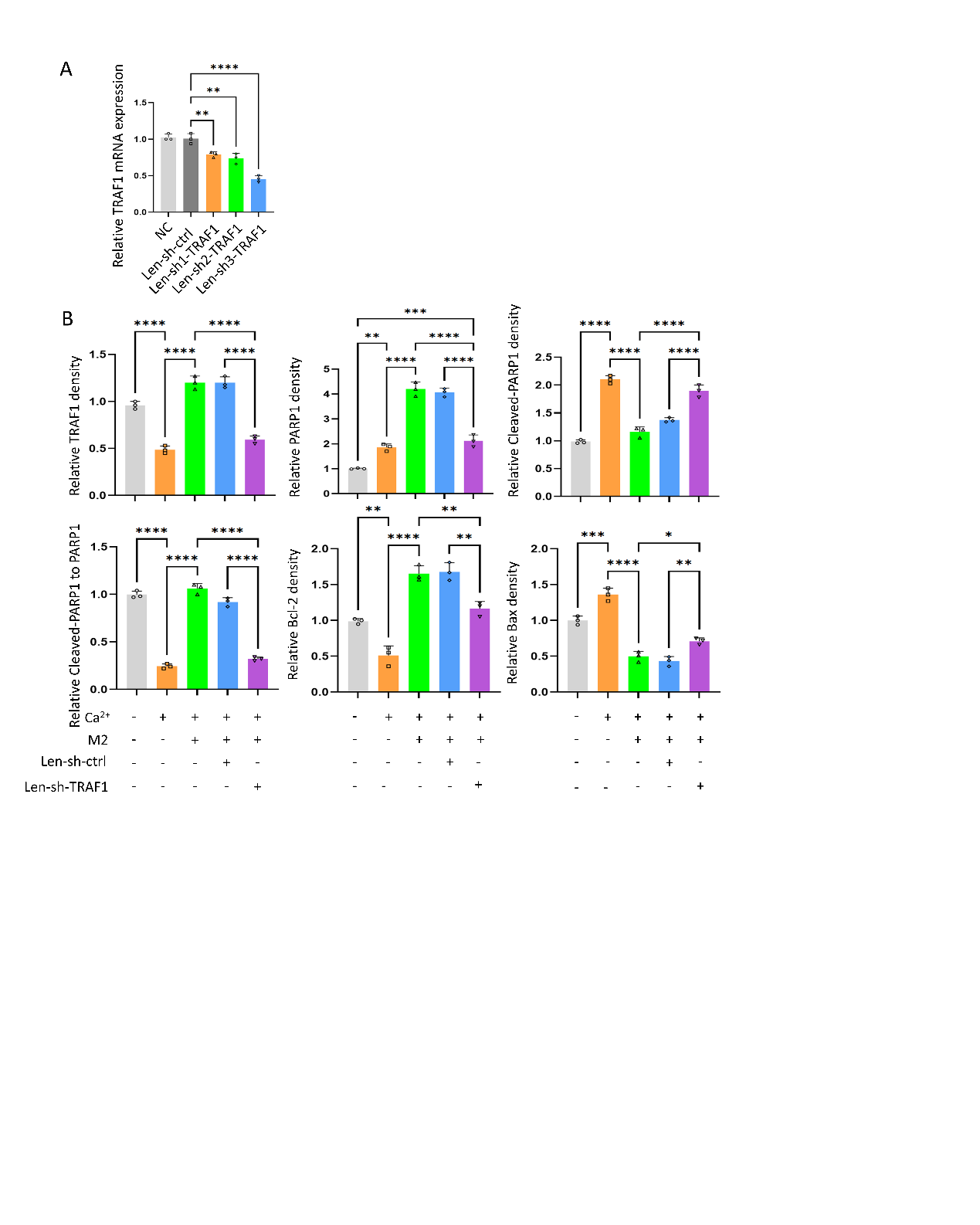


**Figure S3.**

**(A)** TRAF1 knockdown in AQP2⁺ cells was evaluated by qRT-PCR; Len-sh3-TRAF1 showed the highest efficiency and was used for further experiments (n=3).

**(B)** Quantification of PARP1, cleaved-PARP1, Bcl-2 and Bax protein levels by Western blot in TRAF1-knockdown AQP2⁺ cells (n=3).


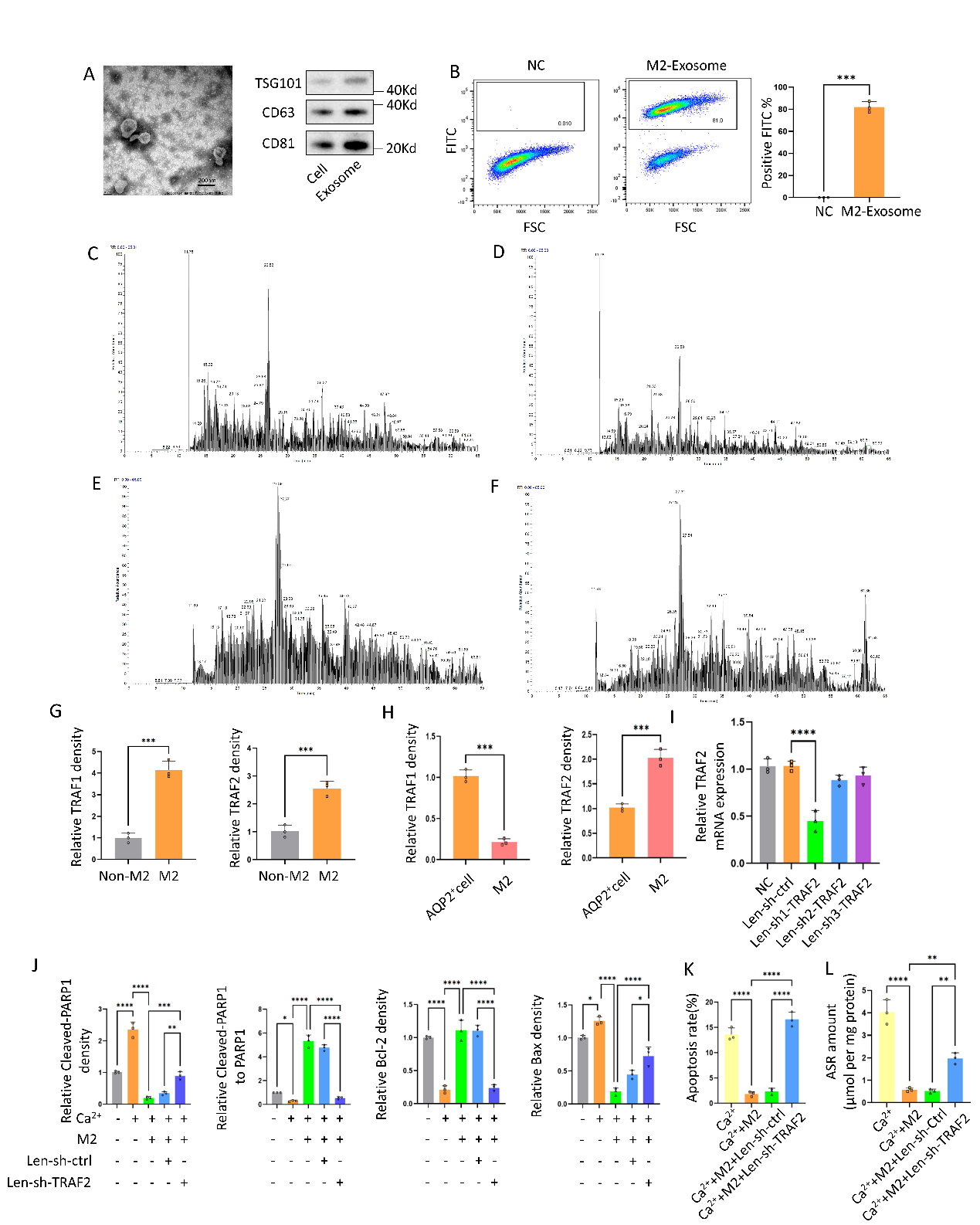


**Figure S4.**

**(A)** Transmission electron microscopy image of M2 macrophage–derived exosome, and Western blotting of exosomal markers (CD63, CD81, and TSG101).

**(B)** DiL (10 μM)-labeled exosomes (20 μg/mL) of M2 macrophages were incubated with AQP2⁺ cells for 48h, and quantitative exosome uptake assays were determined by flow cytometry (n=3).
**(C-D)** Total ion current (TIC) chromatograms of TRAF1 Co-IP mass spectrometry analysis in exosomes isolated from conditioned media of AQP2⁺cells with or without M2 co-culture.
**(E-F)** TIC chromatograms of TRAF1 Co-IP mass spectrometry analysis in AQP2-positive cells with or without M2 co-culture.
**(G)** Quantification of TRAF1 and TRAF2 by Western blot in AQP2⁺ cells cultured alone or co-cultured with M2 macrophage(n=3).

**(H)** Quantification of TRAF1 and TRAF2 by Western blot in comparison between AQP2⁺ cells and M2 macrophage(n=3)
**(I)** TRAF2 knockdown in M2 macrophage was evaluated by qRT-PCR; Len-sh1-TRAF2 showed the highest efficiency and was used for further experiments(n=3).
**(J-L)** Quantification of PARP, cleaved-PARP, Bcl-2, and Bax protein levels by Western blot (J), apoptosis by flow cytometry (K), and calcium deposition by Alizarin Red staining (L) in AQP2⁺ cells co-cultured with M2 or TRAF2-knockdown M2 macrophage(n=3).


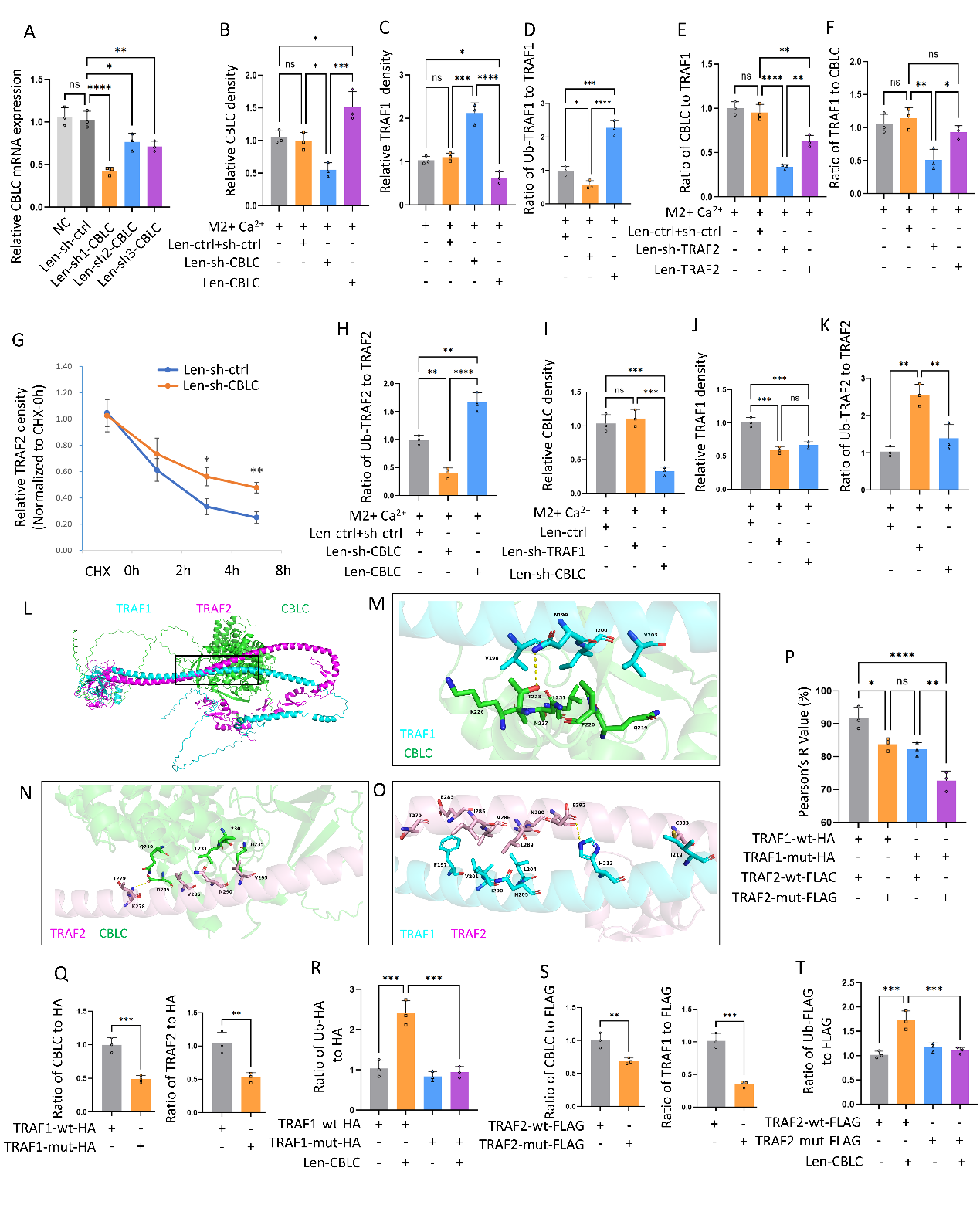


**Figure S5**

**(A)** CBLC knockdown in AQP2⁺ cells was confirmed by qRT-PCR.

**(B-D)** Quantification of CBLC expression, TRAF1 expression, and TRAF1 ubiquitination in AQP2⁺ cells with CBLC overexpression or knockdown.

**(E-F)** Quantification of CBLC–TRAF1 interaction by Co-IP in AQP2⁺ cells with TRAF2 overexpression or knockdown.

**(G)** Quantification of TRAF2 protein levels by Western blot at 0, 2, 4, and 8 h after CHX treatment in CBLC-knockdown AQP2⁺ cells.

**(H)** Quantification of Ub-TRAF2/TRAF2 levels by Western blot in AQP2⁺ cells with CBLC overexpression or knockdown.

**(I-K)** Quantification of CBLC, TRAF1, and Ub-TRAF2/TRAF2 levels by Western blot in AQP2⁺ cells with CBLC or TRAF2 knockdown.

**(L-O)** Cartoon model of the interaction interface of TRAF1-CBLA, TRAF2-CBLC, and TRAF1-TRAF2 predicted by AlphaFold3.

**(P)** Co-localization of TRAF1 and TRAF2was quantified using Pearson’s correlation coefficient (R) calculated by ImageJ.

**(Q)** Quantification of TRAF1–CBLC and TRAF1–TRAF2 interactions by Co-IP in AQP2⁺ cells with TRAF1 mutation.

**(R)** Quantification of Ub-TRAF1/TRAF1 levels by Western blot in CBLC-overexpressing AQP2⁺ cells with TRAF1 mutation.

**(S)** Quantification of TRAF2–CBLC and TRAF2–TRAF1 interactions by CO-IP in AQP2⁺ cells with TRAF1 mutation.

**(T)** Quantification of Ub-TRAF2/TRAF2 levels by Western blot in CBLC-overexpressing AQP2⁺ cells with TRAF2 mutation.


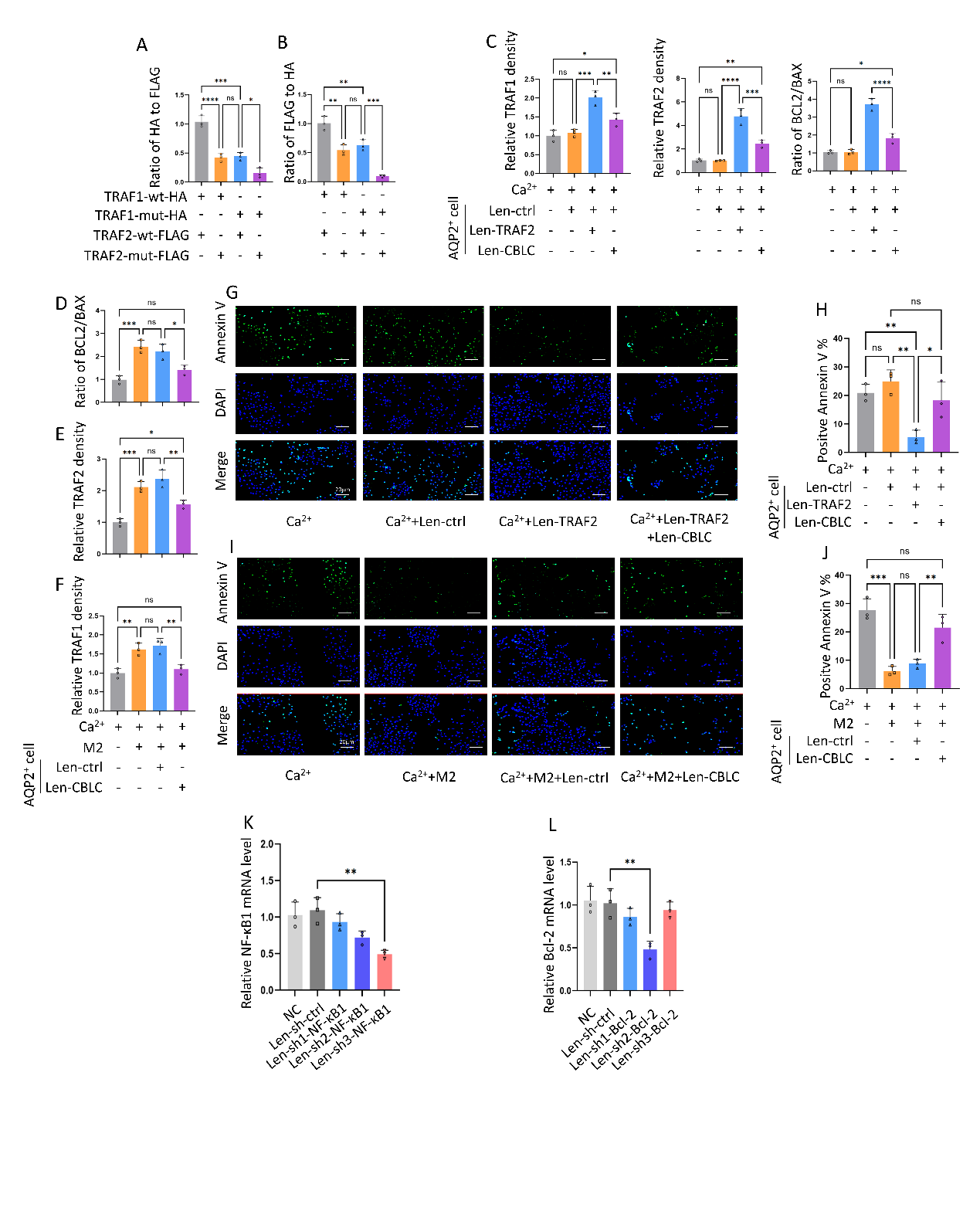


**Figure S6.**

**(A-B)** Quantification of TRAF1–TRAF2 interaction by co-immunoprecipitation in AQP2⁺ cells with individual or combined TRAF1/TRAF2 mutations.

**(C)** Quantification of TRAF1 and TRAF2 protein levels, and ratio of BCL2/BAX by Western blot in AQP2⁺ cells overexpressing TRAF2 or CBLC.

**(D-F)** Quantification of TRAF1 and TRAF2 protein levels, and ratio of BCL2/BAX by Western blot in CBLC-overexpressing AQP2⁺ cells co-cultured with M2 macrophages.

**(G-H)** Apoptosis was examined by Annexin V staining in AQP2⁺ cells overexpressing CBLC or TRAF2.

**(I-J)** Apoptosis was examined by Annexin V staining in CBLC-overexpressing AQP2⁺ cells co-cultured with M2 macrophages.

**(K)** NF-κB1 knockdown in AQP2^+^ cells was evaluated by qRT-PCR; Len-sh3-NF-κB1 showed the highest efficiency and was used for further experiments.

**(L)** Bcl-2 knockdown in AQP2^+^ cells was evaluated by qRT-PCR; Len-sh2-NF-κB1 showed the highest efficiency and was used for further experiments.


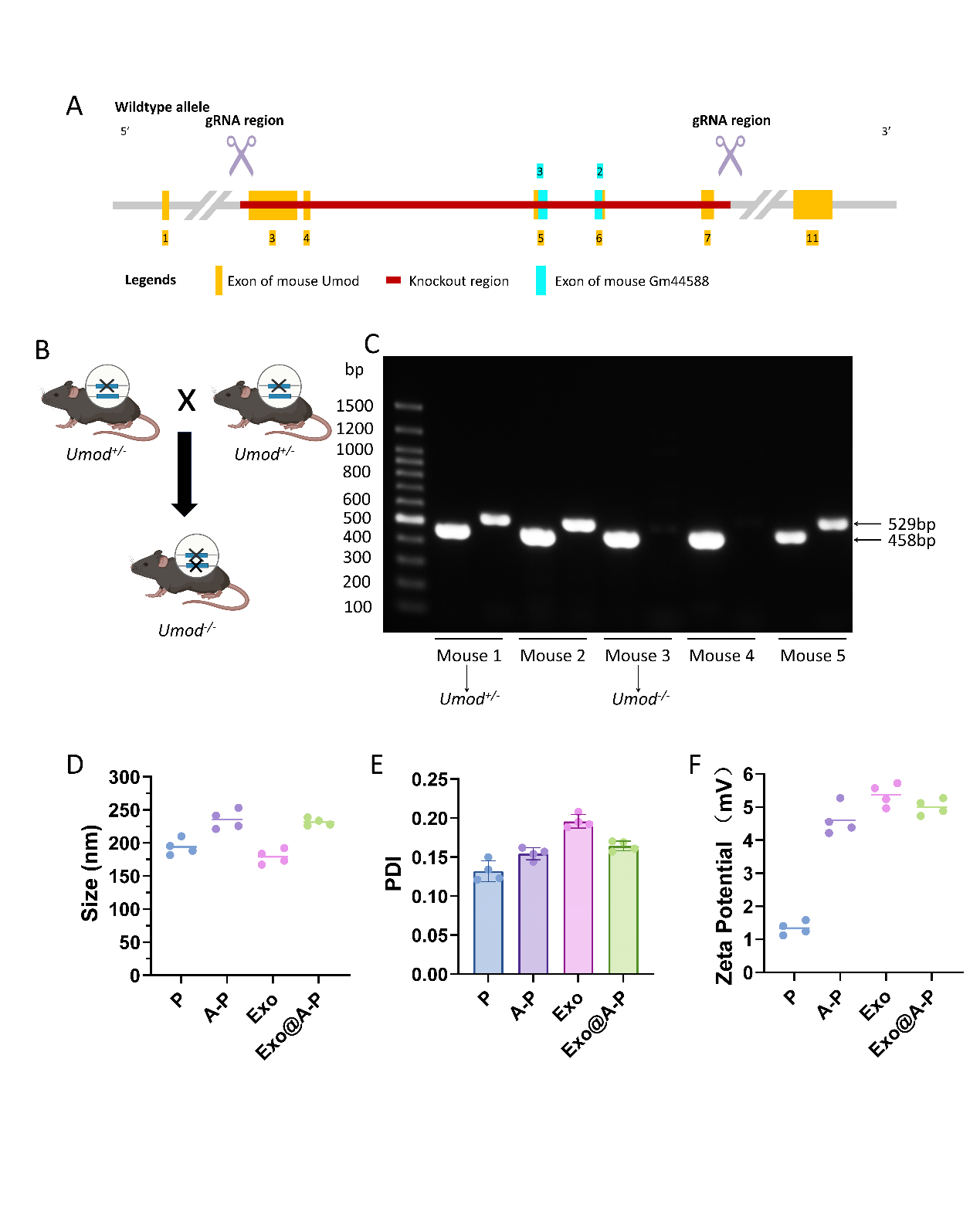


**Figure S7.**

**(A)** Schematic diagram illustrating generation of germline Umod^-/-^ mice. Exon 3-7 was selected as the knockout region.

**(B)** The mating scheme used to generate Umod^-/-^ mice.

**(C)** Genotyping PCR products of littermates from the mating of heterozygous mice.

**(D)** Particle size analysis of P, A-P, Exo and Exo@A-P formulations measured by dynamic light scattering. n=4 for each group.

**(E)** Polydispersity index (PDI) of P, A-P, Exo and Exo@A-P formulations. n=4 for each group.

**(F)** Zeta potential analysis showing the surface charge variations among P, A-P, Exo, and Exo@A-P. n=4 for each group.


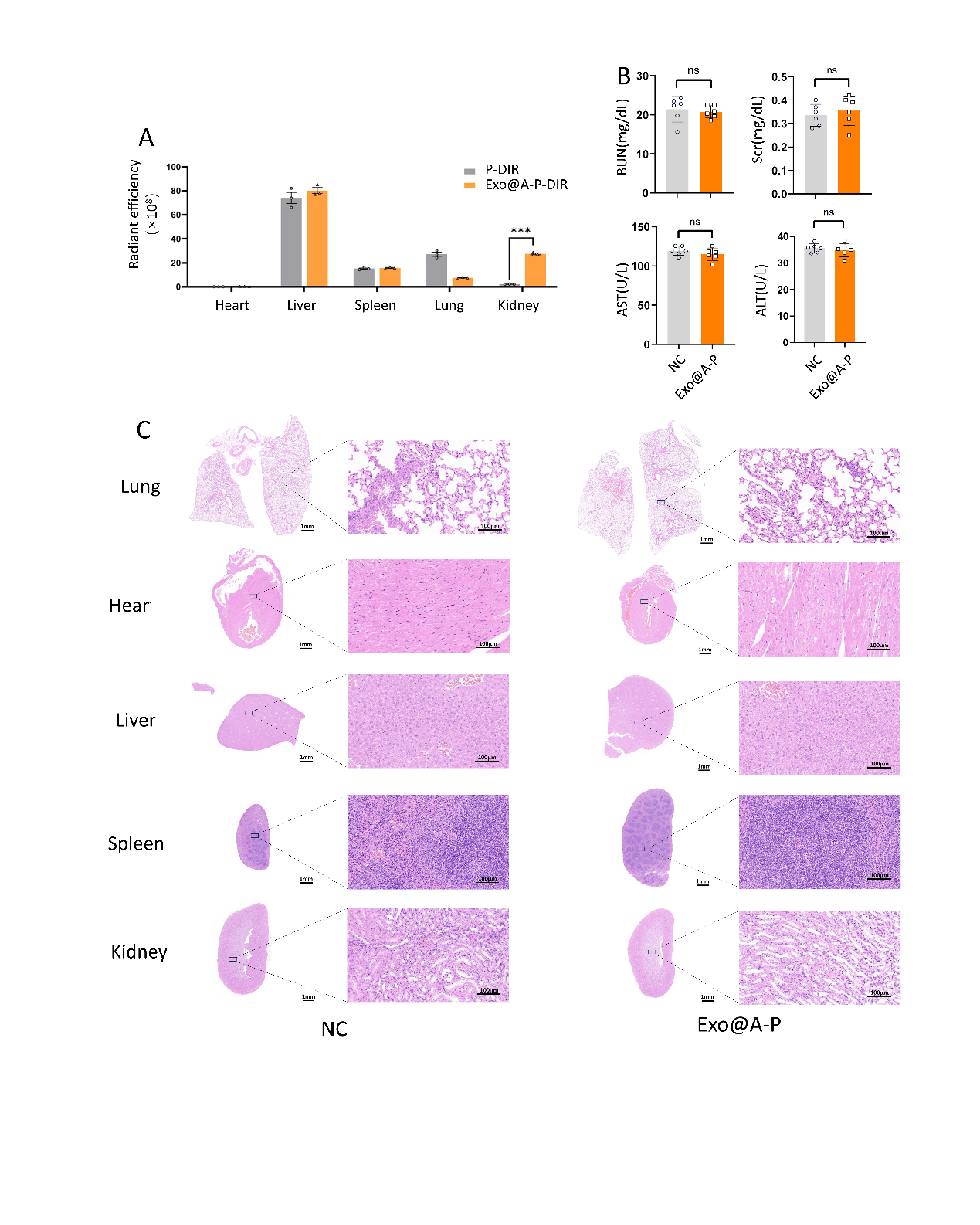


**Figure S8.**

**(A)** Quantification of Ex vivo fluorescence intensity in major organs harvested 48 hours post-injection (n=3).

**(B)** Biochemical analysis of serum collected from mice in the NC and Exo@A-P treatment groups. Blood urea nitrogen (BUN) and serum creatinine (Scr) were measured to assess kidney function, and aspartate aminotransferase (AST) and alanine aminotransferase (ALT) levels were measured to evaluate liver function. n=6 for each group.

**(C)** H&E staining of lung, heart, liver, spleen and kidney tissues from mice treated with exosome-loaded AQP2⁺ cell membrane–coated PLGA nanoparticles (Exo@A-P) (n=6) and control groups (n=6).


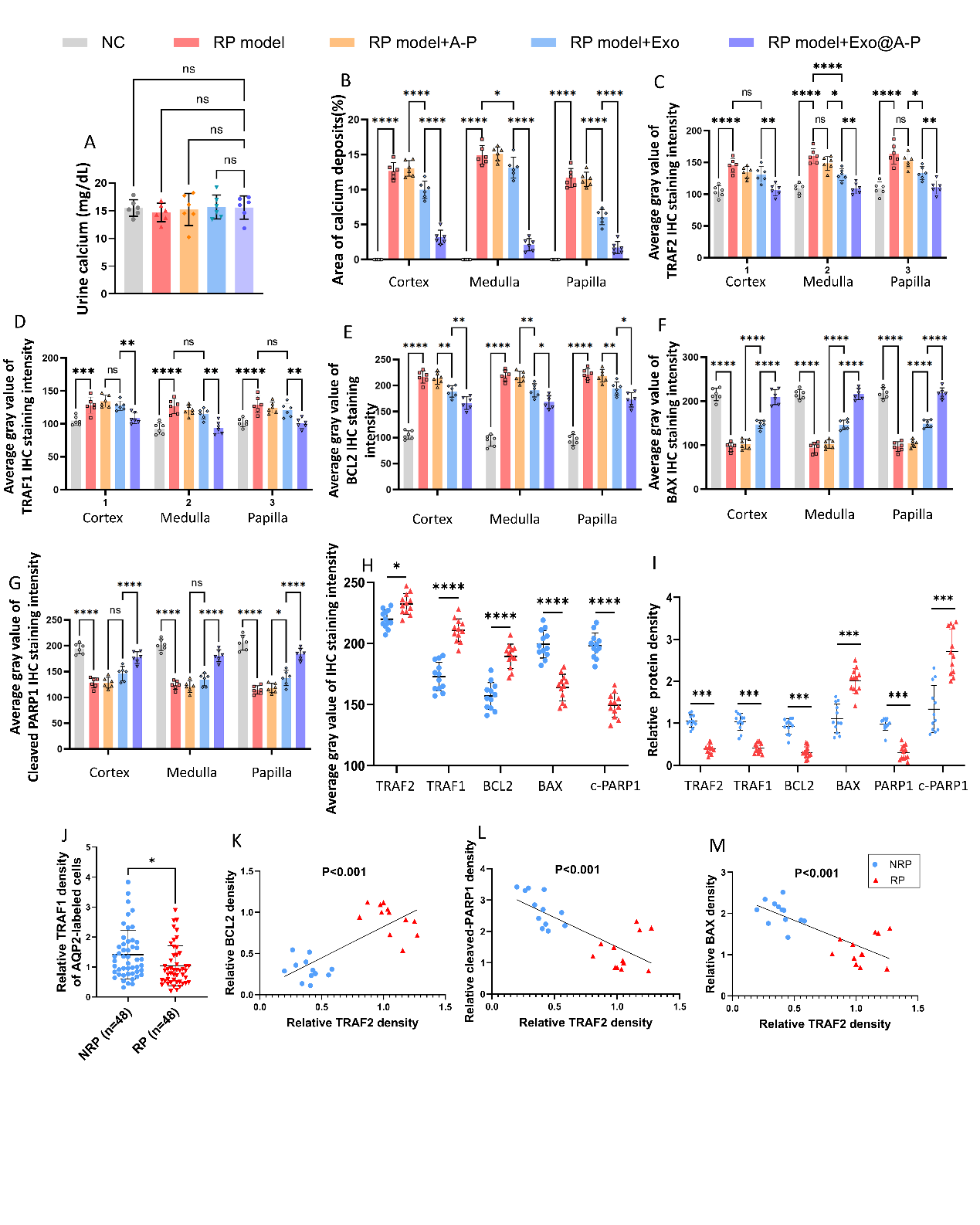


**Figure S9.**

**(A)** Calcium excretion of 24h urine across different treatment groups. n=6 for each group.

**(B)** Von Kossa staining in mouse kidneys across different treatment groups. n=6 for each group.

**(C-G)** Quantification of IHC Immunohistochemical staining of TRAF2, TRAF1, Bcl-2, Bax and Cleaved-PARP1 in mouse kidneys across different treatment groups. n=6 for each group. The average grey value was assigned on a scale from 0 to 254, where 0 represented black, dark-stained areas and 254 represented white, unstained areas. Consequently, DAB staining intensity exhibited an inverse correlation with the average grey value.

**(H)** Quantification of TRAF1, TRAF2, BCL2, BAX, PARP1 and Cleaved-PARP1 determined by immunohistochemical staining in human NRP (n=12) and RP (n=12) tissues.

**(I)** Quantification of TRAF1, TRAF2, BCL2, BAX, PARP1 and Cleaved-PARP1 determined by WB in human NRP (n=12) and RP (n=12) tissues.

**(J)** Relative TRAF1 density of AQP2-labeled cells determined by the immunofluorescence staining in a tissue microarray carrying 48 pairs of NRP and RP tissues.

**(K-M)** Correlation analysis of TRAF2 and BCL2; BAX; cleaved-PARP1 identified by WB in NRP (n=12) and RP (n=12) tissues.

**Supplementary references**

1. Zhu, Z., et al., *Osteogenic-Like Microenvironment of Renal Interstitium Induced by Osteomodulin Contributes to Randall's Plaque Formation.* Adv Sci (Weinh), 2024. **11**(40): p. e2405875.

2. Shen, K., et al., *GRP78 expression in tumor and perinephric adipose tissue is not an optimal risk stratification marker for clear cell renal cell carcinoma.* PLoS One, 2019. **14**(1): p. e0210246.

3. Shu, J., et al., *Statistical colour models: an automated digital image analysis method for quantification of histological biomarkers.* Biomed Eng Online, 2016. **15**: p. 46.

4. Wolf, F.A., P. Angerer, and F.J. Theis, *SCANPY: large-scale single-cell gene expression data analysis.* Genome Biol, 2018. **19**(1): p. 15.

5. Korsunsky, I., et al., *Fast, sensitive and accurate integration of single-cell data with Harmony.* Nat Methods, 2019. **16**(12): p. 1289–1296.
